# Intrinsic myocardial defects underlie an Rbfox-deficient zebrafish model of hypoplastic left heart syndrome

**DOI:** 10.1038/s41467-022-32982-x

**Published:** 2022-10-05

**Authors:** Mengmeng Huang, Alexander A. Akerberg, Xiaoran Zhang, Haejin Yoon, Shakchhi Joshi, Celia Hallinan, Christopher Nguyen, William T. Pu, Marcia C. Haigis, C. Geoffrey Burns, Caroline E. Burns

**Affiliations:** 1 Division of Basic and Translational Cardiovascular Research, Department of Cardiology, Boston Children’s Hospital, Boston, MA 02115 USA; 2 Harvard Medical School, Boston, MA 02115 USA; 3 Department of Cell Biology, Blavatnik Institute, Harvard Medical School, Boston, MA 02115 USA; 4 Cardiovascular Research Center, Massachusetts General Hospital, Charlestown, MA 02129 USA; 5 Athinoula A Martinos Center for Biomedical Imaging, Charlestown, MA 02129 USA; 6 Harvard Stem Cell Institute, Cambridge, MA 02138 USA; 7 Present Address: Department of Biological Sciences, Ulsan National Institute of Science and Technology, Ulsan, 44919 Republic of Korea; 8 Present Address: Cardiovascular Innovation Research Center, Heart Vascular & Thoracic Institute, Cleveland Clinic, Cleveland, OH 44194 USA

**Keywords:** Disease model, Development, Cardiovascular diseases

## Abstract

Hypoplastic left heart syndrome (HLHS) is characterized by underdevelopment of left sided structures including the ventricle, valves, and aorta. Prevailing paradigm suggests that HLHS is a multigenic disease of co-occurring phenotypes. Here, we report that zebrafish lacking two orthologs of the RNA binding protein *RBFOX2*, a gene linked to HLHS in humans, display cardiovascular defects overlapping those in HLHS patients including ventricular, valve, and aortic deficiencies. In contrast to current models, we demonstrate that these structural deficits arise secondary to impaired pump function as these phenotypes are rescued when Rbfox is specifically expressed in the myocardium. Mechanistically, we find diminished expression and alternative splicing of sarcomere and mitochondrial components that compromise sarcomere assembly and mitochondrial respiration, respectively. Injection of human *RBFOX2* mRNA restores cardiovascular development in *rbfox* mutant zebrafish, while HLHS-linked *RBFOX2* variants fail to rescue. This work supports an emerging paradigm for HLHS pathogenesis that centers on myocardial intrinsic defects.

## Abstract (web summary)

The developmental mechanisms responsible for the structural defects observed in hypoplastic left heart syndrome remain controversial. Using rbfox-deficient zebrafish, the authors implicate impaired cardiac function as a primary driver of disease.

## Introduction

Hypoplastic left heart syndrome (HLHS) is a severe form of congenital heart disease (CHD) that is characterized by underdevelopment of left-sided structures including the left ventricle (LV), mitral and aortic valves, and ascending aorta^1,2^. Without treatment, HLHS is uniformly lethal due to inadequate systemic circulation. Consequently, three palliative surgeries are performed to recruit the right ventricle (RV) as the sole pumping chamber. While a single ventricle (SV) physiology allows HLHS patients to survive, the long-term prognosis is poor with only 36–50% of patients living without a transplant due to ventricular dysfunction^3–5^. Currently, there is no way to stratify the patient population into those who will respond well to the palliative surgery versus those who will develop early heart failure^2,6^.

A genetic basis for HLHS is indicated by increased risk of recurrence in families^7,8^, yet the specific genes affected remain largely undefined. This lack of genetic information can be attributed in part to the rarity of the disease and its complex pattern of inheritance^1,2^. Prior failures to recover animal models of HLHS supports the idea of a multigenic etiology^2,9^. In fact, the only existing animal model recovered from a forward genetic screen, termed the *Ohia* mouse line, has a digenic origin with mutations in *Sap130* causing LV deficiencies that are separable from *Pcdha9*-induced aortic valve defects^10^. No genetic model has been described where a single gene mutation causes the full spectrum of HLHS cardiovascular phenotypes including ventricular, valve, and aortic deficiencies.

HLHS patients have an increased burden of damaging de novo variants in *RBFOX2*, which encodes a highly conserved RNA binding protein with known roles in splicing^11,12^. While statistical enrichment of *RBFOX2* mutations in HLHS is highly significant, and therefore suggestive of causality, this hypothesis has yet to be tested. Arguing against pathogenicity, mice with *Rbfox2* deleted in *Mlc2v*+ cardiomyocytes are born with grossly normal hearts^13^. However, a recent report demonstrated that earlier deletion of *Rbfox2* in *Nkx2.5*+ cells, which include cardiac progenitors, is embryonic lethal by E11.5^14^. Specifically, these animals are severely growth impeded and display gross morphological defects in cardiac chamber, common outflow tract, and yolk sac vasculature development. Consistent with these findings, morpholino-mediated knock-down of two RBFOX2 orthologs in zebrafish, Rbfox1-like (Rbfox1l) and Rbfox2, induces gross morphological and functional cardiac defects^15^. However, these zebrafish phenotypes were not investigated in detail and have not been confirmed in genetic mutants. As such, it remains unclear how mutations in Rbfox2 affect cardiovascular development.

Here, we used CRISPR-Cas9 genome editing to create zebrafish lacking Rbfox1l and Rbfox2. We find that 100% of double mutant embryos succumb by 4 days post-fertilization (dpf) from HLHS-like malformations that develop subsequent to a functional deficiency, which are associated with compromised sarcomere assembly and mitochondrial respiration. We also report that injection of wildtype human *RBFOX2* mRNA rescues the cardiovascular defects present in *rbfox*-deficient zebrafish, while HLHS-linked *RBFOX2* variants fail. These data demonstrate that the molecular functions of RBFOX2 proteins are conserved between human and zebrafish and provide genetic evidence that *RBFOX2* mutations are causal for HLHS pathogenesis. Overall, our work supports an emerging paradigm where structural defects associated with HLHS can manifest downstream of impaired cardiac pump function.

## Results

### Rbfox1l and Rbfox2 function redundantly to support cardiovascular and skeletal muscle development in zebrafish

**Fig. 1:**
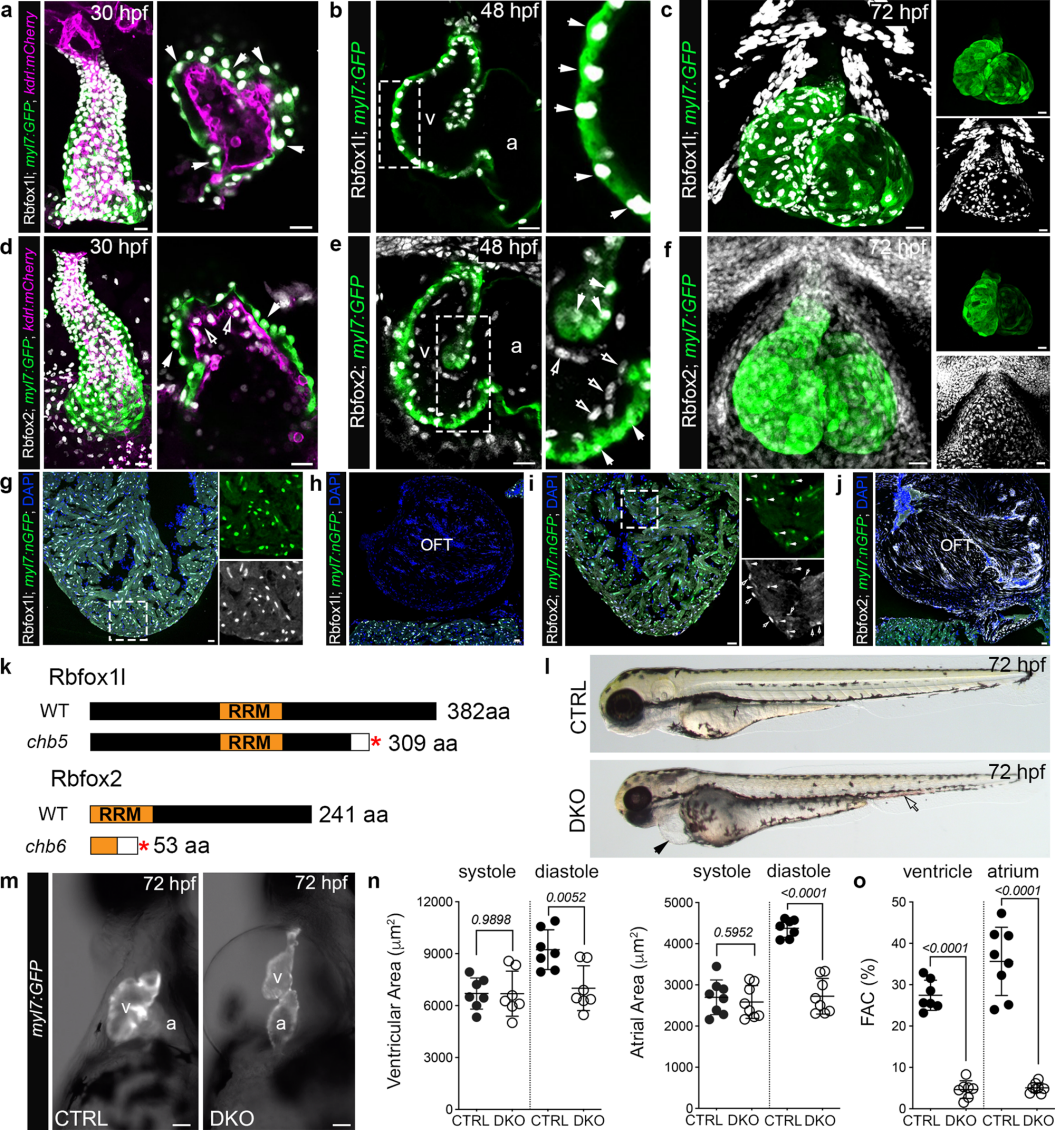
Rbfox1l and Rbfox2 function redundantly to support cardiovascular development in zebrafish. **a**–**f** Confocal z-stacks and single optical sections of 30, 48, and 72 hpf hearts from *Tg(myl7:GFP);Tg(kdrl:mCherry)* zebrafish following immunostaining for GFP, mCherry, Rbfox1l, and Rbfox2 as indicated. White arrows show Rbfox1l or Rbfox2 within cardiomyocytes. Open arrows show Rbfox2 in endocardial cells. Boxed regions are shown at higher magnification. Split channels of (**c**) and (**f**) are shown. **g**–**j** Merged confocal images of cardiac sections from *Tg(myl7:nGFP)* adult zebrafish following immunostaining for GFP, Rbfox1l, and Rbfox2 and counterstained with DAPI. Boxed regions in **g** and **i** are shown as split channels. White arrowheads show Rbfox2 in cardiomyocytes. Open arrowheads show Rbfox2 in presumptive endocardial cells. **k** Diagrams of wildtype Rbfox1l and Rbfox2 and the predicted protein products of the *rbfox1l*^*chb5*^ and *rbfox2*^*chb6*^. Red asterisks mark the locations of premature stop codons. White boxes indicate divergent amino acids. **l** Brightfield images of 72 hpf CTRL and DKO embryos. Black arrowhead highlights pericardial edema. Open arrowhead highlights pooled blood. **m** Brightfield images with fluorescent overlay of *Tg(myl7:GFP)* signal in hearts from 72 hpf CTRL and DKO embryos. **n**, **o** Dot plots showing chamber areas during systole and diastole and percent fractional area change (%FAC) in 72 hpf CTRL and DKO hearts. Sample sizes and Statistics: (**a**–**j**) Little to no variation in expression was observed. *n* = 10 embryos/developmental stage/condition; *n* = 6 adults/condition. **n**, **o** Each dot represents one embryo in all graphs. Data are presented as mean values +/− one SD. Statistical significance was determined by an unpaired, two-tailed Student’s *t*-test assuming equal variances. *P* values are shown. Source data are provided as a Source Data file. Abbreviations: v ventricle, a atrium, OFT outflow tract, aa amino acid, RRM RNA-recognition motif. Scale bars: 20 μm.

To gain spatial resolution into the reported cardiac expression of *rbfox1l* and *rbfox2*^15^, we immunostained double transgenic embryos carrying the myocardial *Tg(myl7:GFP)* and endothelial *Tg(kdrl:mCherry)* fluorescent reporters with previously described antibodies that recognize Rbfox1l and Rbfox2^16^. While Rbfox1l is restricted to GFP+ cardiomyocyte nuclei at linear heart tube and chamber stages (Fig. 1a–c), Rbfox2 localizes to both myocardial and endocardial nuclei including the developing endocardial cushions, which give rise to the valve in the atrioventricular canal (AVC; Fig. 1d–f). At 72 h post-fertilization (hpf), Rbfox1l remains restricted to striated muscle (Fig. 1c) while Rbfox2 is more broadly distributed (Fig. 1f). In the adult heart, Rbfox1l co-localizes with GFP in myocardial nuclei (Fig. 1g and Supplementary Fig. 1a) with no obvious expression in the outflow tract (OFT; Fig. 1h), the equivalent of the aortic root in mammals^17^. Rbfox2 predominantly localizes to endocardial cells, relatively fewer cardiomyocytes, and smooth muscle cells of the OFT (Fig. 1i, j and Supplementary Fig. 1b). Based on their overlapping cardiac expression, we hypothesized that Rbfox1l and Rbfox2 function redundantly to support zebrafish cardiogenesis.

To test this hypothesis, we used CRISPR-Cas9 to create deleterious mutations in each locus. We isolated a 14 base pair deletion in exon 8 of *rbfox1l* (*rbfox1l*^*chb5*^*)* and a 2 base pair deletion in exon 3 of *rbfox2* (*rbfox2*^*chb6*^*)* that shift the open reading frame and introduce a premature stop codon (Fig. 1k). Transcripts for *rbfox1l* and *rbfox2* are reduced by approximately 50% or greater, respectively, in *rbfox1l*^*−/−*^*; rbfox2*^*−/−*^ double knock-out (referred to hereafter as DKO) embryos compared to controls (Supplementary Fig. 1c), demonstrating that the mutations undermine transcript production and/or stability. Next, we immunostained single *rbfox2* mutants for Rbfox1l and single *rbfox1l* mutants for Rbfox2 and found that each protein is expressed as predicted (Supplementary Fig. 1d). Moreover, we learned that DKO hearts are completely devoid of Rbfox1l and Rbfox2 (Supplementary Fig. 1d), revealing that *chb5* and *chb6* are null alleles.

Although single mutants do not exhibit overt cardiac phenotypes during embryogenesis, DKO animals display paralysis, pericardial edema, and poor circulation as evidenced by blood pooling in the tail (Fig. 1l). By 3 days post-fertilization (dpf), 100% of DKO embryos exhibit severe morbidity and succumb by 4–5 dpf. To examine heart function, still frames were extracted from videos of GFP-expressing control and DKO hearts over successive cardiac cycles (Fig. 1m; Supplementary Movie 1 and 2) and used to quantify chamber areas during systole and diastole. Although ventricular and atrial areas are similar between cohorts during systole, a 32% and 38% reduction was observed in DKO during diastole, respectively (Fig. 1n). To estimate heart function, these area measurements were used to calculate percent fractional area change (%FAC), which approached zero in both DKO chambers (Fig. 1o). Together, these data are consistent with required and redundant functions for Rbfox1l and Rbfox2 in cardiovascular development in zebrafish.

### *Rbfox*-deficient zebrafish display cardiovascular defects that mirror those in HLHS

**Fig. 2:**
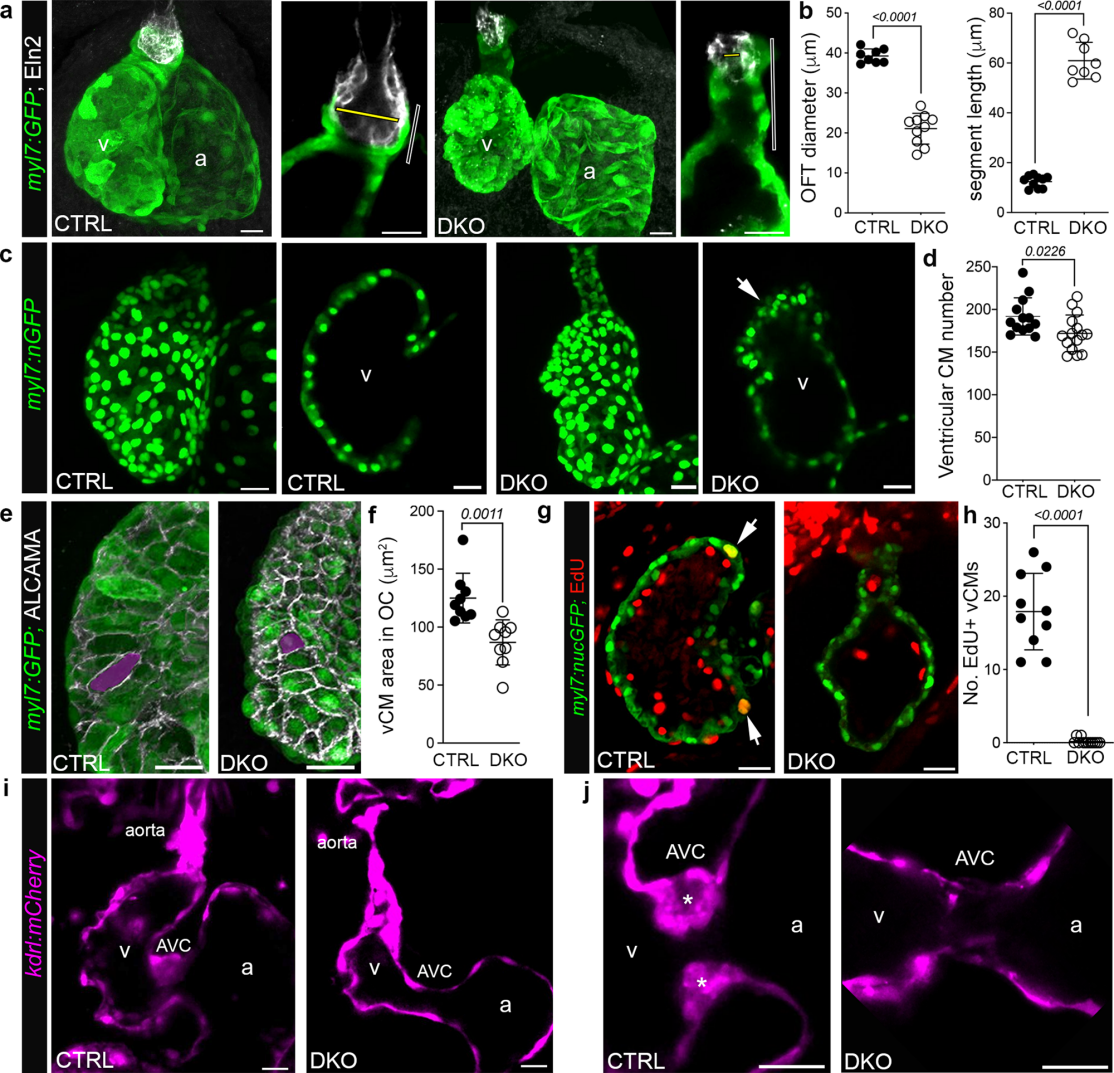
*Rbfox*-deficient zebrafish display cardiovascular defects overlapping those in HLHS hearts. **a** Confocal z-stacks or single optical sections of hearts or OFTs from 72 hpf CTRL or DKO embryos carrying the Tg(*myl7:GFP)* reporter following immunostaining for GFP (green) and Elastin2 (Eln2; white). Yellow bars, OFT diameter. Open bars, myocardial segment length. **b** Dot plots showing OFT diameter and myocardial segment length. **c** Confocal z-stacks or single optical sections of hearts from 72 hpf CTRL and DKO embryos carrying the *Tg*(*myl7:nGFP)* transgene following immunostaining for GFP (green). Arrow highlights myocardial disorganization in the DKO ventricle. **d** Dot plot of ventricular cardiomyocyte numbers. **e** Confocal z-stacks of the ventricular OC from 72 hpf CTRL and DKO embryos carrying the *Tg(myl7:GFP)* reporter following immunostaining for GFP (green) and ALCAMA (white). Magenta overlay shows the area of a representative OC cardiomyocyte. **f** Dot plot showing the average area of ventricular OC cardiomyocytes. **g** Single optical section of the ventricle from 72 hpf CTRL and DKO embryos carrying the *Tg(myl7:nGFP)* transgene following immunostaining for GFP (green) and EdU (red). White arrows highlight GFP+; EdU+ cardiomyocytes. **h** Dot plot showing the number of EdU+ ventricular cardiomyocytes. **i** Single optical sections of hearts from live 72 hpf CTRL and DKO embryos carrying the *Tg*(*kdrl:mCherry)* transgene. **j** Single optical sections of the AVC. Asterisks mark endocardial cushions. Sample Sizes and Statistics: (**b**, **d**, **h**) Each dot represents one embryo. **f** Each dot represents the average area of 8 outer curvature cardiomyocytes from 8 CTRL and 10 DKO hearts. Data are presented as mean values +/− one SD. Statistical significance was determined by an unpaired, two-tailed Student’s *t*-test assuming equal variances. *P* values are shown. Source data are provided as a Source Data file. Abbreviations: v ventricle, a atrium, CM cardiomyocyte, OC outer curvature, AVC atrioventricular canal. Scale bars: 20 μm.

Because deleterious mutations in *RBFOX2* are known to segregate with HLHS^11,12^, we assessed DKO hearts for phenotypic characteristics associated with the disease. Confocal z stacks of control and DKO hearts carrying the *Tg(myl7:GFP)* reporter following immunostaining for GFP and Elastin2b (Eln2), a marker of smooth muscle cells in the OFT, confirmed the presence of a diminutive ventricle with an abnormal myocardial segment that is significantly narrower and longer in DKO compared to controls (Fig. 2a, b). The restriction of Eln2+ smooth muscle cells to the distal-most region of the segment suggests that this relatively small portion represents the OFT while the adjacent section is part of the ventricular chamber that failed to balloon. We confirmed this interpretation by prospective lineage tracing of *nkx2.5*+ progenitors in pharyngeal arch 2 that give rise to OFT but not ventricular myocardium^18^ (Supplementary Fig. 2a–f). Using this boundary information, we quantified ventricular cardiomyocyte numbers at 72 hpf and discovered significantly fewer in DKO that appear highly unorganized in the distal wall (Fig. 2c, d). In addition, ventricular cardiomyocytes are 44% smaller in the outer curvature (Fig. 2e, f) and fail to proliferate as demonstrated by a lack of EdU incorporation between 48 and 72 hpf (Fig. 2g, h). In addition to these myocardial deficiencies, the endothelial lining of the aorta appears partially or fully obstructed (Fig. 2i) and the endocardial cushions that give rise to the atrioventricular (AV) valves are completely lacking (Fig. 2j). Collectively, these data demonstrate that Rbfox mutant zebrafish display the cardinal cardiovascular phenotypes associated with HLHS including ventricular, aorta, and valve deficiencies.

### Impaired myocardial contractility occurs prior to other cardiovascular phenotypes in Rbfox-deficient embryos

**Fig. 3:**
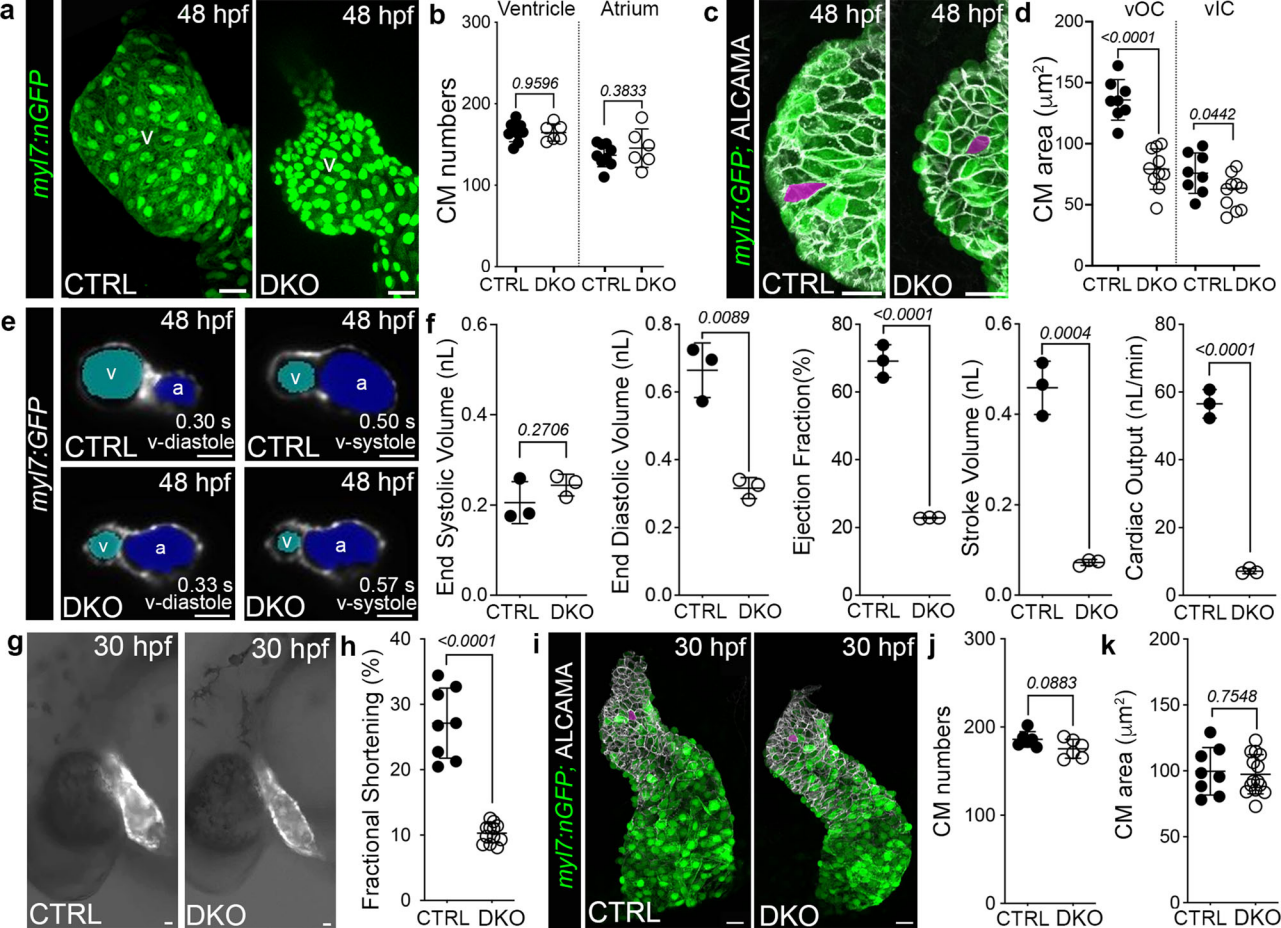
Impaired myocardial function is the earliest cardiovascular defect observed in Rbfox-deficient embryos. **a** Confocal projections of 48 hpf CTRL and DKO hearts carrying the *Tg*(*myl7:nGFP)* transgene immunostained for GFP (green). **b** Dot plot showing cardiomyocyte numbers. **c** Confocal projections of 48 hpf ventricular OCs from CTRL and DKO embryos carrying the *Tg*(*myl7:GFP)* reporter immunostained for GFP (green) and ALCAMA (white). Magenta overlay of OC cardiomyocytes. **d** Dot plot showing the average area of ventricular OC and IC cardiomyocytes. **e** Single-plane images of 48 hpf CTRL and DKO embryos carrying the *Tg(myl7:GFP)* transgene. Atrial (blue) and ventricular (cyan) lumens are shown. **f** Dot plots showing ventricular end-systolic volume (ESV) and end-diastolic volume (EDV) measured with CFIN that were used to calculate ejection fraction (EF), stroke volume (SV), and cardiac output (CO). **g** Brightfield images with fluorescent overlay of *Tg(myl7:GFP)* reporter signal in 30 hpf CTRL and DKO embryos. **h** Dot plot showing % fractional shortening at the arterial pole. **i** Confocal projections of 30 hpf CTRL and DKO hearts carrying the Tg(*myl7:nGFP)* transgene immunostained with anti-GFP (green) and anti-ALCAMA (white). Magenta overlay of arterial pole cardiomyocytes. **j**, **k** Dot plots showing cardiomyocyte numbers and average cell areas. Sample sizes and Statistics: (**b**, **f**, **h**, **j**) Each dot represents one embryo. **d** Each dot represents the average area of 8 OC cardiomyocytes and 6 IC cardiomyocytes from 8 CTRL and 10 DKO hearts. **k** Each dot represents the average area of 8 cardiomyocytes from 8 CTRL and 12 DKO hearts. Data are presented as mean values +/− one SD. Statistical significance was determined by an unpaired, two-tailed Student’s *t*-test assuming equal variances. *P* values are shown. Source data are provided as a Source Data file. Abbreviations: v ventricle, a atrium, OC outer curvature, IC inner curvature. Scale bars: 20 μm.

To learn which of the cardiovascular phenotypes are observed first, we analyzed heart structure and function in control and DKO embryos carrying the *Tg(myl7:nGFP)* reporter at 48 hpf, a developmental stage that precedes myocardial proliferation and valve formation. Confocal Z-stacks revealed diminutive ventricular chambers composed of equivalent numbers of cardiomyocytes that are significantly smaller in size (Fig. 3a–d). These data demonstrate that reductions in cardiomyocyte cell growth initially account for the reduced ventricular chamber area, a phenotype that is later exacerbated by decreased proliferation. To quantify functional parameters at 48 hpf, ventricular chamber volumes were measured in serial images of control and DKO hearts carrying the *Tg(myl7:GFP)* reporter over successive cardiac cycles by light sheet fluorescent microscopy (LSFM) and processed using our machine learning platform Cardiac Functional Imaging Network (CFIN; Fig. 3e)^19^. Although end systolic volumes (ESV) are not different between cohorts, end diastolic volumes (EDV) are reduced by 52% in DKO ventricles, which manifests as significant decreases in ejection fraction (EF), stroke volume (SV), and cardiac output (CO) (Fig. 3f). Strikingly, this functional deficit is observed even earlier at the arterial pole of the linear heart tube (Fig. 3g, h), which is prior to reductions in cardiomyocyte cell numbers or area (Fig. 3i–k). Because this contractile phenotype precedes formation of the cardiac chambers, aorta, and valves, we conclude that Rbfox proteins play a primary role in promoting myocardial pump function.

### Rbfox proteins are required for expression and alternative splicing of transcripts encoding mitochondrial and sarcomere components

**Fig. 4:**
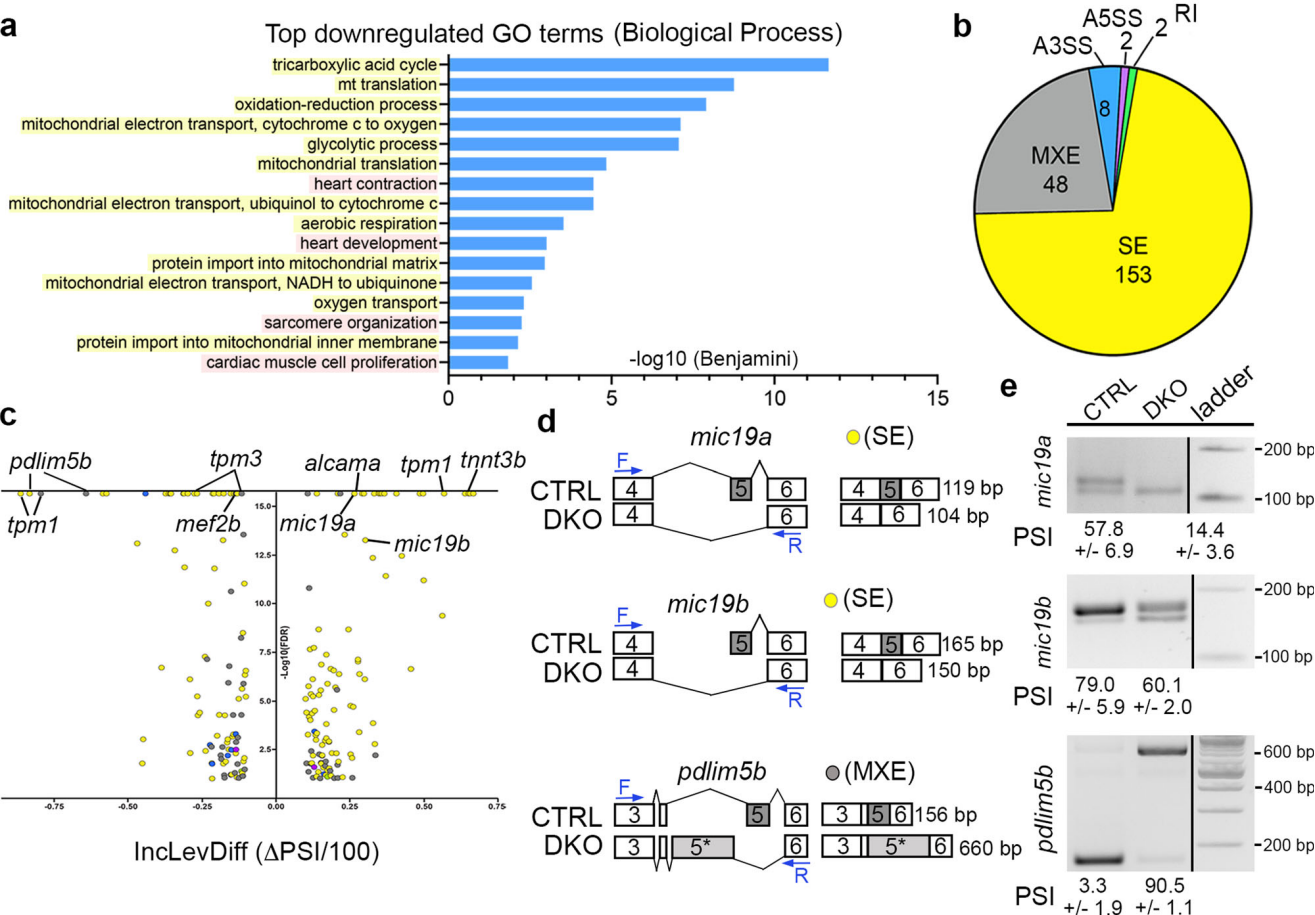
Rbfox proteins are required for expression and alternative splicing of transcripts encoding mitochondrial, cytoskeletal, and sarcomere components. **a** GO analysis showing biological processes associated with downregulated transcripts in DKO hearts. Yellow represents mitochondria-associated terms, while pink shows heart muscle terms. **b** Pie chart depicting the proportions of differential alternative splicing events in each category. Abbreviations: Skipped exon (SE), mutually exclusive exon (MXE), alternative 3’ splice site (A3SS), alternative 5’ splice site (A5SS), and retained intron (RI). **c** Volcano plot showing the inclusion level difference (IncLevDiff; CTRL Inclevel - DKO Inclevel) and False Discovery Rates (FDR) for differential alternative splicing events (0 uncalled replicates; FDR < 0.01; |Incleveldifference | >0.1). **d** Schematic representing differential alternative splicing events of select transcripts between CTRL and DKO hearts. Blue arrows define primer sites used in the RT-PCR assay to calculate percent spliced in (PSI) shown in (**e**). **e** RT-PCR validation of 3 alternative splicing events identified by rMATS. PSI is shown. Sample sizes and Statistics: For RNA-sequencing, *n* = 8 biological independent replicates per cohort with 35–70 pooled hearts per replicate.

To uncover the molecular mechanisms leading to the observed functional deficiencies, we performed bulk RNA-sequencing on isolated hearts from 48 hpf control and DKO embryos. Through differential expression analysis, we identified 563 downregulated and 421 upregulated transcripts (Supplementary Table 1). Genes downregulated in DKO hearts were enriched for gene ontology (GO) biological process terms associated with mitochondrial function and sarcomere assembly (Fig. 4a, Supplementary Table 2). Specifically, we identified transcripts in mitochondrial protein import (e.g., *samm*, *mppα*, *tomm, timm*, and *pam* complex members), which function to shuttle nuclear-encoded proteins through the mitochondrial membrane for use in oxidative phosphorylation^20^. Reduced expression of MICOS complex components (*mic19b*, *mic25*, and *mic60*), which create the mitochondrial inner membrane folds where the electron transport chain functions, were also observed^21^. Downregulation of subunits of the electron transport chain itself were also identified (e.g., *nduf, cox*, *uqcr*) along with nearly all of the mitochondrial ribosomal protein subunits (termed *mrpl* and *mrps*). Although the vast majority of GO terms involve mitochondrial function, diminished expression of prominent sarcomere components (e.g., *cmlc1*, *tpm4a*, *tnni4a*, *myh7l*, and *acta1b*) were also identified (Fig. 4a). We confirmed downregulation of several transcripts in each GO category by real-time quantitative RT-PCR (Supplementary Fig. 3a).

Because RBFOX proteins are known to regulate RNA splicing^22^, we performed replicate multivariate analysis of transcript splicing (rMATS)^23^ and discovered 213 significant alternative splicing events in DKO hearts (Supplementary Table 3). The most common event was skipped exon (SE), representing over 71% of the total, followed by mutually exclusive exon (MXE, 22%), alternative 3’ splice site (A3SS, 5%), alternative 5’ splice site (A5SS, 1%), and retained intron (RI, 1%) (Fig. 4b, Supplementary Table 3). Alternative splicing events with the highest significance and inclusion level differences were detected in transcripts encoding MICOS complex components (e.g. *mic19a, mic19b*), sarcomere components (e.g. *tpm1, tpm3, tnnt3b*), and cytoskeletal components (e.g. *pdlim5b, alcama, fmnl3*) (Fig. 4c; Supplementary Table 3). In the case of *mic19a* and *mic19b*, which encode the zebrafish orthologs of the essential MICOS complex component MIC19^21,24^, inclusion of exon 5 was significantly reduced in DKO hearts when compared to controls (Fig. 4d; Supplementary Fig. 3b; Supplementary Table 3). For *pdlim5b*, a pair of mutually exclusive exons (17 and 542 nucleotides) were alternatively spliced in DKO hearts to favor the inclusion of the longer exon (Fig. 4d, e; Supplementary Fig. 3b; Supplementary Table 3). To validate these results, we used RT-PCR to quantify percent spliced in (PSI) for isoform-specific exons between 48 hpf control and DKO hearts for *mic19a*, *mic19b*, and *pdlim5b* (Fig. 4e). In addition, we used real-time quantitative RT-PCR to determine relative levels of total transcript and alternatively spliced isoforms between cohorts for *mic19a, mic19b, pdlim5b*, and *tpm1* (Supplementary Fig. 3b). In agreement with our RNAseq results, total transcript levels were decreased for *mic19a* and *pdlim5b*, while no significant difference was observed for *mic19a* or *tpm1* (Supplementary Fig. 3b) However, relative isoform-specific transcript levels were significantly changed for all events tested with several exhibiting over a three-fold difference between cohorts (Supplementary Fig. 3b). Taken together, these molecular alterations suggest that defects in sarcomere assembly and mitochondrial respiration might contribute to the ventricular size and functional deficiencies observed in DKO hearts.

### Rbfox proteins are required for sarcomere assembly and mitochondrial metabolism

**Fig. 5:**
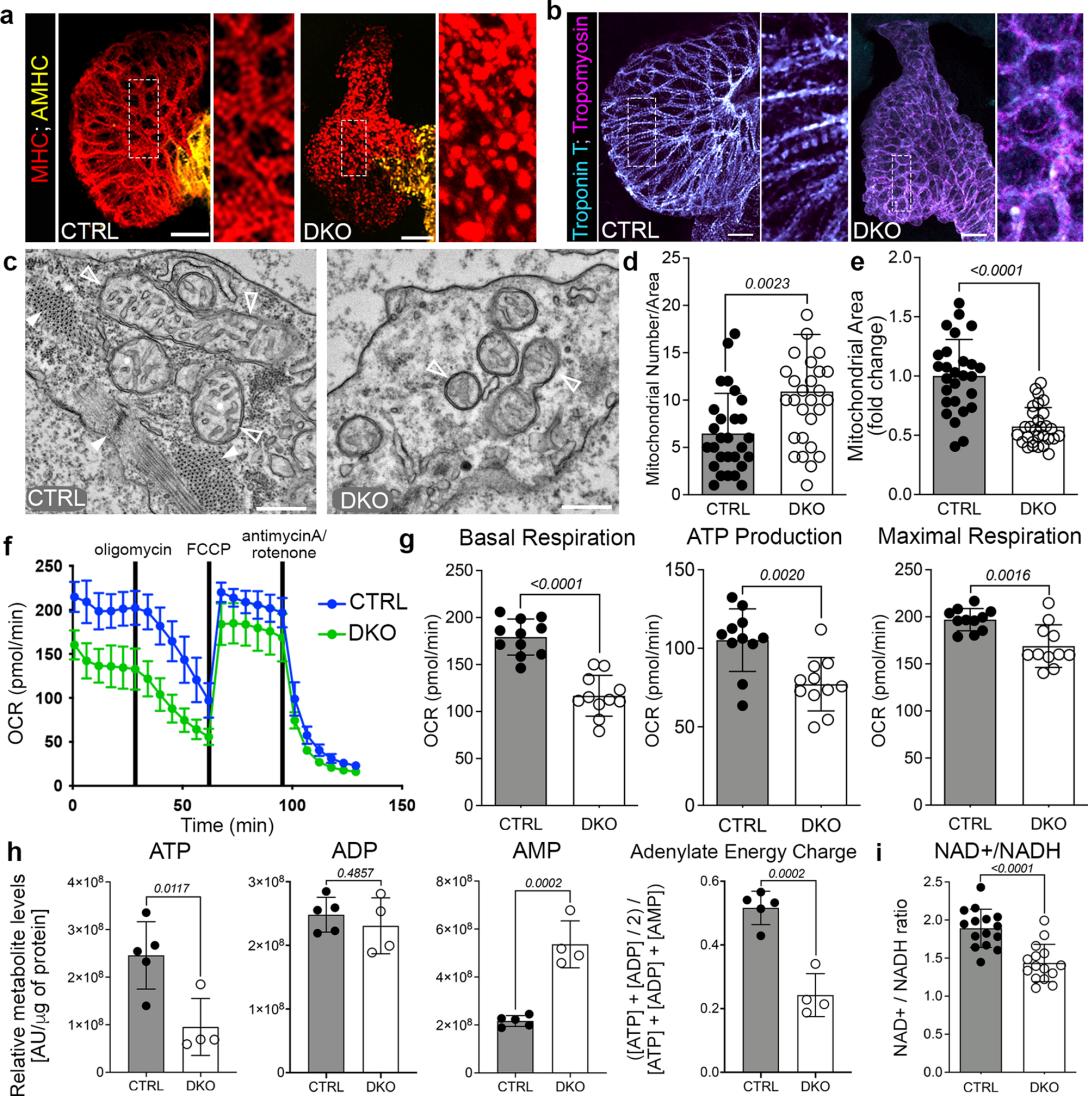
Rbfox proteins mediate sarcomere assembly and mitochondrial respiration. **a**, **b** Merged confocal projections of 48 hpf CTRL or DKO hearts immunostained for MHC and AMHC (**a**), and Troponin T and Tropomyosin (**b**). Magnified views of boxed regions are shown. **c** Transmission electron micrographs (TEMs) of ventricular cardiomyocytes in 48 hpf CTRL or DKO. White arrowheads highlight sarcomere bundles and a Z-line in CTRL. Open arrowheads highlight mitochondria. **d**, **e** Dot plots depicting mitochondrial numbers per area and fold change in mitochondrial area. **f** Graph of seahorse mito stress test to measure oxygen consumption rates (OCR) of 48 hpf CTRL or DKO embryos. Drug addition is indicated. **g** Dot plots from raw data in **f**. **h** Dot plots of metabolomics data in 24 hpf CTRL or DKO embryos. **i** Dot plot showing NAD+/NADH ratio at 48 hpf by GloAssay. Sample sizes and statistics: (**a**, **b**) Little to no variation in expression was observed. *n* = 10 embryos/cohort. **c** *n* = 3 embryos/cohort. **d**, **e** Each dot represents the number of mitochondria per 8000X section and fold change in mitochondrial area (CTRL: 11 sections from Heart 1, 7 sections from Heart 2, and 9 sections from Heart 3; DKO: 8 sections from Heart 1, 10 sections from Heart 2, and 11 sections from Heart 3). **f** *n* = 3 biologically independent replicates with one representative graph shown. Each dot in (**f**) represents 11 embryos per cohort. **h** *n* = 4 CTRL or 5 DKO biological replicates with 40 deyolked 24 hpf embryos/replicate. All metabolites were normalized by protein level. **i** *n* = 3 embryos/dot. Data are presented as mean values+/− one SD. Statistical significance was determined by an unpaired, two-tailed Student’s *t*-test assuming equal variances. *P* values are shown. Source data are provided as a Source Data file. Scale bars: 20 μm.

To test whether these molecular alterations translate into sarcomere and mitochondrial abnormalities, we first compared myofibril architecture between 48 hpf control and DKO hearts. To visualize sarcomere alignment, we performed immunofluorescence with antibodies that recognize Myosin Heavy Chain (MHC), Atrial Myosin Heavy Chain (AMHC), Troponin T, and Tropomyosin. As anticipated, control hearts show highly ordered myofibrils with prominent striations (Fig. 5a, b). Although DKO hearts express the proteins analyzed, striations were absent, demonstrating that the myofibrils did not assemble appropriately (Fig. 5a, b). Transmission electron micrographs confirmed these results as sarcomere bundles and Z-lines were apparent in 48 hpf control ventricular cardiomyocytes but absent from DKO (Fig. 5c). Myofibrillar disarray was also observed in DKO animals at the linear heart tube stage (30 hpf; Supplementary Fig. 4) when cardiac contractions normally commence^25^. These data demonstrate that Rbfox proteins are essential for sarcomere assembly in vivo and suggest that this defect contributes to the functional deficiency observed in DKO hearts.

To test if mitochondrial ultrastructure is affected in DKO cardiomyocytes, we analyzed the same transmission electron micrographs for alterations in mitochondrial morphology. Unlike mitochondria from control cardiomyocytes that have characteristic round or elongated shapes with regularly dispersed cristae, mitochondria from DKO cardiomyocytes appeared malformed with less defined or missing inner membrane folds (Fig. 5c). This notable ack of discernable cristae is consistent with downregulation and alternative splicing of *mic19*, which has been shown to be essential for normal mitochondrial morphology, cristae distribution, and ATP production^24^. Upon quantification, we found that there were significantly more mitochondria per area in DKO cardiomyocytes (Fig. 5d), but they were significantly smaller in size (Fig. 5e).

To determine if mitochondrial respiration is affected, we performed a mitochondrial stress test using the Agilent Seahorse analyzer to determine oxygen consumption rates (OCR) in individual control or DKO embryos at 48 hpf (Fig. 5f). Basal respiration, ATP production, and maximal respiration were all significantly reduced in *rbfox* mutant embryos (Fig. 5g), demonstrating that mitochondrial respiration is compromised in the absence of Rbfox activity. As an alternative approach, we performed untargeted metabolomics on 24 hpf control and DKO embryos (Supplementary Table 4). Although a low number of metabolites were detected in our samples, likely reflecting low sample input, we observed significantly reduced levels of ATP, no change in ADP, and a significant increase in AMP (Fig. 5h; Supplementary Table 4). These data are consistent with failure to generate ATP during oxidative phosphorylation. To quantify the overall energy status of control and DKO cells, we calculated the Adenylate Energy Charge using the following formula: ([ATP] + ½ [ADP])/([ATP] + [ADP] + [AMP]) and found a significant reduction in DKO embryos (Fig. 5h). Finally, the NAD+/NADH ratio, which is directly linked to oxygen consumption rate and mitochondrial metabolism, was also significantly decreased in 48 hpf DKO animals compared to controls (Fig. 5i). Taken together, these data demonstrate that Rbfox proteins are essential for sarcomere assembly, mitochondrial ultrastructure, electron transport chain gene expression, and ATP production, which when defective compromises cardiomyocyte pump function and cell growth.

### Myocardial-specific expression of Rbfox1l rescues ventricular structure and function, valve development, and aortic stenosis in DKO hearts

**Fig. 6:**
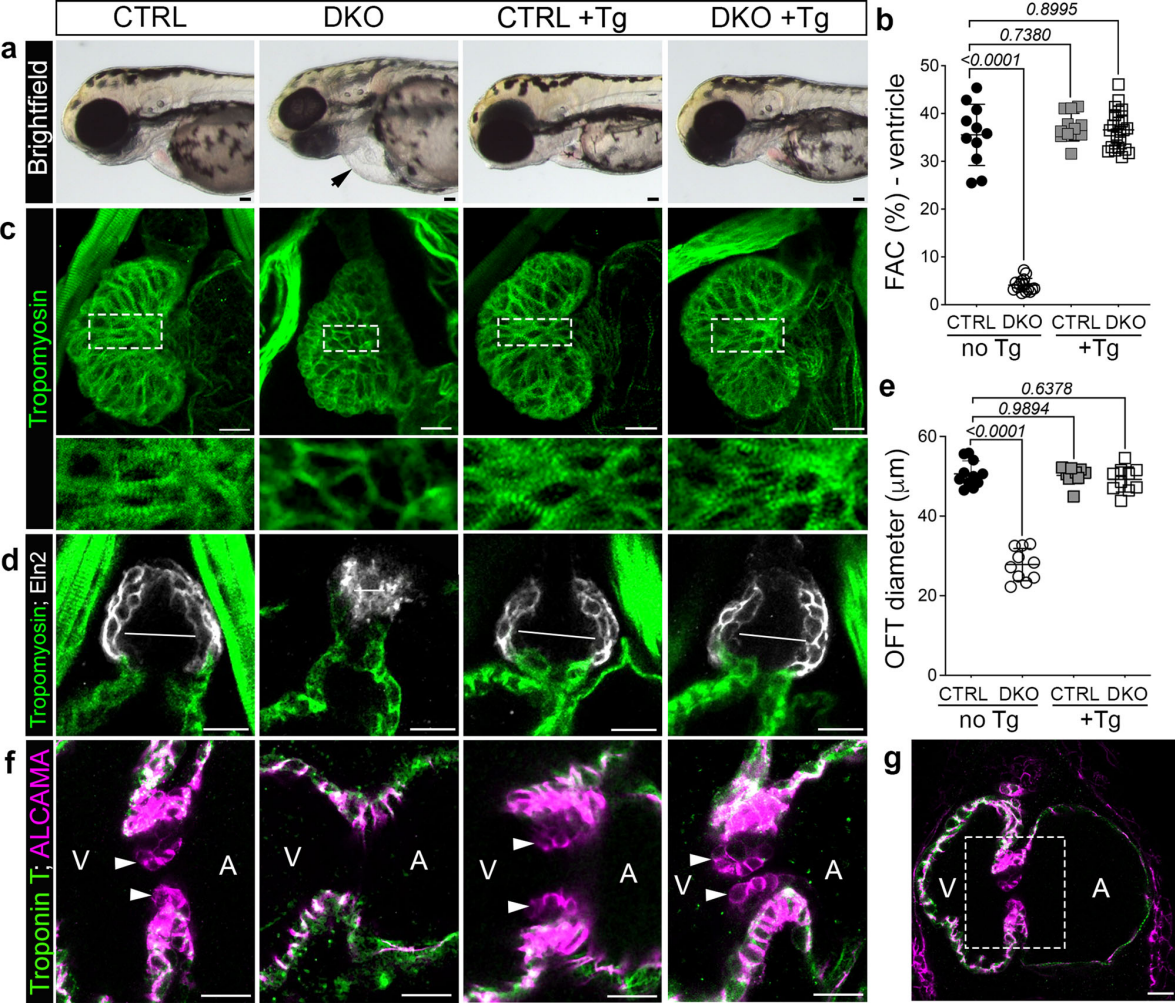
Aortic stenosis and valve atresia in *rbfox*-deficient zebrafish arise secondary to impaired pump function. **a** Brightfield images of 72 hpf CTRL or DKO embryos in the absence or presence of the *Tg(myl7:rbfox1l)* transgene (Tg). Black arrow highlights pericardial edema in DKO. **b** Dot plot showing % fractional area change (FAC) of ventricles in 72 hpf CTRL or DKO embryos in the absence or presence of the *Tg(myl7:rbfox1l*) transgene. **c** Confocal projections of hearts in 72 hpf CTRL or DKO embryos in the absence or presence of the *Tg(myl7:rbfox1l*) transgene following immunostaining for Tropomyosin. Boxed regions are shown below each panel at higher magnification. **d** Confocal sections of outflow tracts (OFTs) in 72 hpf CTRL or DKO embryos in the absence or presence of the *Tg(myl7:rbfox1l*) transgene following immunostaining for Tropomyosin (green) and Eln2 (white). White lines depict OFT diameter. **e** Dot plot showing quantification of OFT diameter. **f** Confocal sections of the atrioventricular canal in 72 hpf CTRL or DKO embryos in the absence or presence of the *Tg(myl7:rbfox1l*) transgene after immunostaining for Troponin T (green) and ALCAMA (pink). White arrowheads mark the endocardial cushions that are lacking DKO. **g** Boxed region approximates the areas shown at higher magnification in **f**. Sample sizes and Statistics: (**a**, **b**) *n* = 11 CTRL, *n* = 15 DKO, *n* = 12 CTRL+ Tg, *n* = 23 DKO+ Tg; (**c**) *n* = 10/cohort; (**d**, **e**): *n* = 11 CTRL, *n* = 10 DKO, CTRL + Tg, DKO + Tg (**f**) *n* = 9 CTRL+ or – the transgene and *n* = 6 DKO+ or – the transgene. Each dot represents 1 embryo in both graphs. Data are presented as mean values +/− one SD. Statistical significance was determined by ordinary one-way ANOVA Dunnet’s multiple comparisons test. *P* values are shown. Source data are provided as a Source Data file. Scale bars: 20 μm.

Based on the timing of phenotype emergence, our data suggest that defective myocardial pump function is primary while aortic stenosis and valve atresia are secondary. To test this hypothesis directly, we engineered a stable transgenic line constitutively expressing *rbfox1l* under the control of the cardiomyocyte-specific *myl7* promoter [*Tg(myl7:rbfox1l)*]. We chose to express zebrafish Rbfox1l rather than Rbfox2 as its RNA Recognition Motif (RRM) is more highly conserved in length and amino acid sequence with that of human RBFOX2. As expected, we found that DKO embryos displayed the characteristic edema indicative of cardiovascular defects relative to control (Fig. 6a). Transgenic animals were indistinguishable from non-transgenic controls (Fig. 6a), demonstrating that higher than endogenous levels of Rbfox activity in the myocardium does not have deleterious consequences. Remarkably, no cardiac edema was observed in DKO animals carrying the transgene (Fig. 6a), indicating that cardiac function might be restored. Videos of GFP-expressing hearts were used to calculate %FAC as previously described. Transgenic embryos did not show any change in %FAC compared to non-transgenic controls (Fig. 6b). However, %FAC was completely rescued to control levels in DKO embryos in the presence of the transgene (Fig. 6b), demonstrating an autonomous role for Rbfox1l in regulating ventricular pump function. Based on Tropomyosin staining, sarcomere assembly was also rescued in DKO cardiomyocytes overexpressing Rbfox1l (Fig. 6c). Remarkably, we found that both aortic stenosis (Fig. 6d, e) and endocardial cushion atresia (Fig. 6f, g) were fully rescued in DKO hearts when Rbfox1l was specifically expressed in cardiomyocytes. Overall, these data demonstrate that the cardinal HLHS phenotypes observed in DKO embryos arise downstream of and secondary to a myocardial-intrinsic defect.

### Injection of human *RBFOX2* mRNA restores cardiovascular development in *rbfox*-deficient zebrafish, while HLHS-linked *RBFOX2* variants do not

**Fig. 7:**
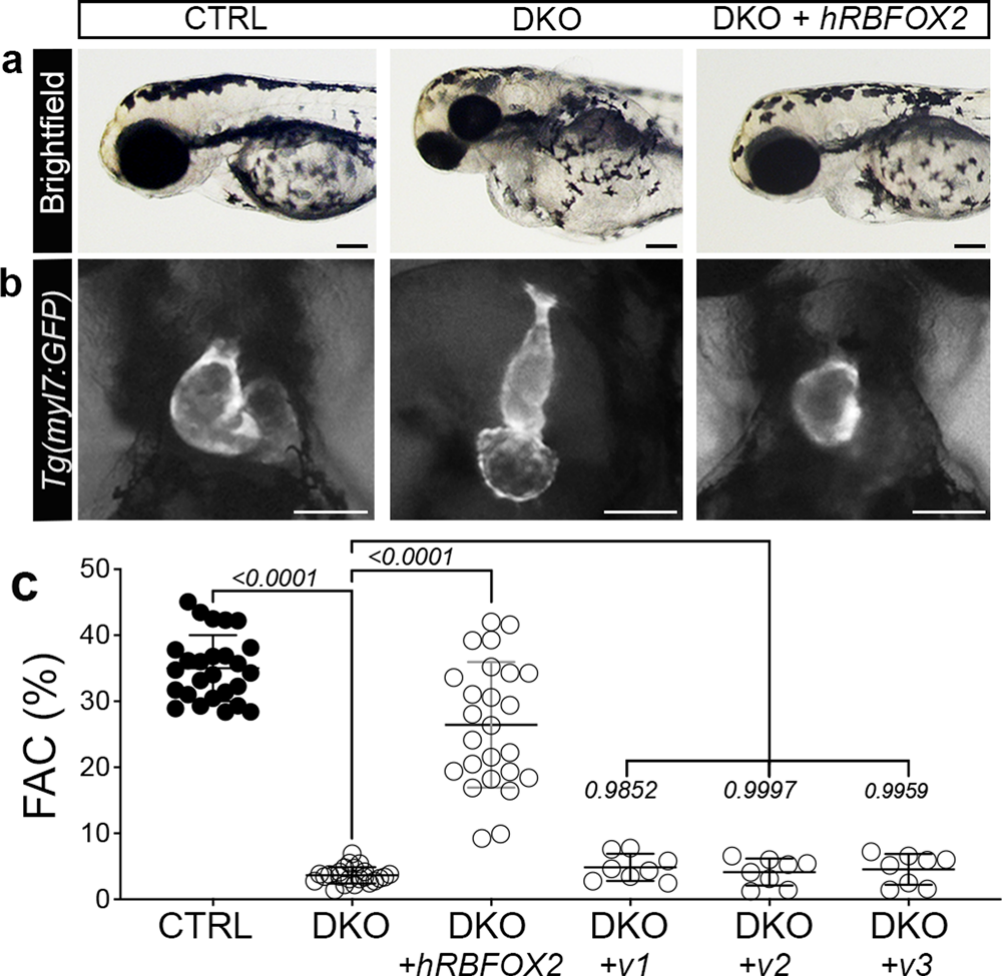
Injection of human *RBFOX2* mRNA restores cardiovascular development in *rbfox*-deficient zebrafish, while HLHS-linked *RBFOX2* variants fail to rescue. **a** Brightfield images of 72 hpf CTRL, DKO, and DKO embryos injected with wildtype human *RBFOX2* (*hRBFOX2*) mRNA. **b** Brightfield images with fluorescent overlay of *Tg(myl7:GFP)* reporter signal in hearts of 72 hpf CTRL, DKO, and DKO embryos injected with wildtype *hRBFOX2* mRNA. **c** Dot plot showing % FAC of 72 hpf ventricles in the following cohorts: uninjected CTRL, uninjected DKO, DKO+ wildtype *hRBFOX2*, DKO+ *RBFOX2-v1, v-2, or v-3* mRNA where *hRBFOX2* harbors a variant (v) previously discovered in patients with HLHS. Sample sizes and statistics: (**c**) uninjected CTRL, *n* = 26; uninjected DKO, *n* = 23; DKO+ wildtype human *RBFOX2*, *n* = 25; DKO + *hRBFOX2 v1, v2*, or *v3* (*n* = 8 per group). Each dot represents 1 embryo. Data are presented as mean values +/− one SD. Statistical significance was determined by ordinary one-way ANOVA Dunnet’s multiple comparisons test. *P* values are shown. Source data are provided as a Source Data file. Scale bars: 20 μm.

To determine if the molecular function of Rbfox proteins is evolutionarily conserved from zebrafish to humans, we attempted to rescue ventricular function in DKO embryos by microinjection of human *RBFOX2* mRNA. As expected, edema was present in uninjected DKO embryos and their hearts were malformed when compared to uninjected controls (Fig. 7a, b). Injection of wildtype human *RBFOX2* mRNA largely suppressed the edema in DKO embryos and restored the heart to a more normal morphology (Fig. 7a, b). Quantification of %FAC revealed that wildtype human *RBFOX2* rescues the contractile deficiency observed in DKO zebrafish, demonstrating conservation of RBFOX2 function between species (Fig. 7c). Unlike wildtype *RBFOX2*, injection of three different variants identified in HLHS patients^11^ failed to restore pump function in DKO hearts (Fig. 7c). These data demonstrate that the cardiac functions of Rbfox proteins are conserved between zebrafish and humans and that these functions are lacking in HLHS-linked variants.

## Discussion

Although whole exome sequencing has revolutionized the discovery of new candidate CHD loci^11,12,26^, follow-up studies in genetically modified animal and iPSC models have been relatively less prolific. Here, we report a new animal model of HLHS that links genetic variants in *RBFOX2* to disease pathogenesis. Moreover, our study demonstrates that while HLHS is thought to be an obligate multigenic disease, it might also arise from a single gene mutation where the structural defects develop secondary to myocardial dysfunction. Overall, our study supports an emerging paradigm for HLHS pathogenesis that centers on myocardial intrinsic abnormalities.

Historically, HLHS was considered a valve disease where the cardinal phenotypes arise from hemodynamic deficiencies^2,27^. Specifically, outflow tract obstruction caused by aortic valve atresia was thought to be a primary driver of HLHS due to reduced blood flow through the LV, which in turn causes secondary chamber growth deficiencies (“no flow, no grow”)^1,27^. This paradigm is consistent with the inheritance of bicuspid aortic valve (BAV), a milder form of aortic valve disease, in first-degree relatives of HLHS probands^28,29^. However, more recent studies connect intrinsic myocardial defects with LV hypoplasia, suggesting that LV and valve phenotypes can arise independently. Several lines of evidence support this claim. Specifically, damaging variants in α-Myosin Heavy Chain *(MYH6)*, which encodes a critical sarcomere component, have been found in individuals with HLHS^30^. Furthermore, iPSC-derived cardiomyocytes from HLHS patients were reported to display either defective differentiation^31^ or impaired contractility^32^ in vitro where hemodynamic forces are lacking. Moreover, myocardial-intrinsic deficiencies were reported in the *Ohia* HLHS mouse model^10^ supporting the idea that LV hypoplasia can manifest from defects within the myocardium. Therefore, it is likely that HLHS can arise through several different mechanisms. As such, linking specific genetic lesions with their primary cellular defect(s) is paramount for developing therapeutic treatment options and for improving the accuracy of prognosis following SV surgery.

In the case of *rbfox* mediated HLHS-like phenotypes in zebrafish, we discovered a primary issue in pump function that leads to compromised development of the valves and aorta. These structural defects are similar to but more severe than those described for two *RBFOX2* heterozygous patients that were reported to have aortic valve stenosis or atresia, mitral valve stenosis, and hypoplasia of the ascending aorta^11^. Why Rbfox-induced defects in myocardial contractility result in HLHS while other mutations that compromise ventricular function do not remains unclear. One possibility is that the combination of decreased sarcomere assembly and mitochondrial respiration synergistically contribute to disease pathogenesis. The independence of these two abnormalities seems likely as we observed decreased expression and alternative splicing of both sarcomere and mitochondrial transcripts in *rbfox*-deficient zebrafish hearts. Consistent with this finding, a previous report documented decreased expression of sarcomere and mitochondrial components in Rbfox2-depleted rat myoblasts due to alternative polyadenylation^33^. However, conditional knock-out of Rbfox2 in the embryonic mouse heart was linked to changes in alternative splicing of transcripts involved in cell adhesion to the extra-cellular matrix^14^, a process not readily identified in our datasets. Therefore, a more comprehensive cross-species comparison of alternative splicing changes caused by loss of Rbfox2 in the heart could be informative. Of particular interest from our study is the identification of decreased expression and alternative splicing of *mic19*, which is essential for mitochondrial ultrastructure and metabolism^21^. Consistent with these findings, iPSC-derived cardiomyocytes from HLHS patients with heart failure also showed significant reductions in mitochondrial metabolism with lower basal respiration and ATP production^32^. By contrast, increased basal respiration was reported in the *Ohia* HLHS mouse heart^6^. These opposing outcomes suggest that differences in disease etiology might account for contrasting effects on cardiomyocyte respiration. Further investigation into the prevalence of metabolic dysregulation across HLHS models will be needed to learn whether this abnormality contributes broadly to myocardial dysfunction. Although it is currently unknown whether *RBFOX2* itself becomes secondarily downregulated in the SV myocardium in the absence of *RBFOX2* mutations, decreased expression could contribute to early heart failure in a broader population and represent a shared pathway to disease pathogenesis.

Taken together, our data highlight that mutations in Rbfox2 orthologs are sufficient to cause the full spectrum of HLHS-like phenotypes in zebrafish through a primary functional deficiency and underscore the need for whole animal modeling of this complicated disease. Based on our expression and alternative splicing data, it is likely that primary defects in both sarcomere assembly and mitochondrial respiration independently contribute to myocardial dysfunction. As such, procedures such as fetal aortic valvuloplasty to reduce outflow tract obstruction^34^ would be unlikely to benefit those with *RBFOX2*-mediated HLHS. Moreover, our data suggest that HLHS patients with *RBFOX2* mutations are more likely to develop heart failure following palliative surgery, suggesting that early transplantation might be critical for this cohort.

## Methods

### Zebrafish husbandry and strains

Zebrafish (*Danio rerio*) were bred and maintained following protocols approved by the Institutional Animal Care and Use Committee (IACUC) of Massachusetts General Hospital and Boston Children’s Hospital. All procedures followed the guidelines and recommendations outlined by the Guide for the Care and Use of Laboratory Animals. The following zebrafish strains were used in this study: *Tg(myl7:GFP)*^*f1*^^35^, *Tg(myl7:nGFP)*^*fb18*^^36^, *Tg(kdrl:mCherry)*^*s896*^^37^, *TgBAC(nkx2.5:Kaede)*^*fb9*^^38^, *rbfox1l*^*chb5*^ (this study), *rbfox2*^*chb6*^ (this study), *Tg(myl7:GFP-P2A-rbfox1l)*^*chb7*^ (this study). Zebrafish strains including *fb* and *chb* alleles that were used in this study are available upon request.

### Generation and genotyping of *rbfox1l*^*chb5*^ and *rbfox2*^*chb6*^ mutant alleles

Guide RNAs targeting the sequence 5ʹ- TGTGGCAGCGGCGGCTG −3ʹ in exon 8 for *rbfox1l* and 5ʹ- GAGATCATCTTCAATGAAAG −3ʹ in exon 3 for *rbfox2* were generated and injected at a final concentration of 150 ng/μl with Cas9 protein (New England Biolabs) into one-cell stage zebrafish embryos as previously described^39^. Germline transmission of CRISPR/Cas9-induced deletions was detected using fluorescent PCR and DNA-fragment analysis as previously described^40^. For *rbfox1l*, 14 bp (CCGCAGCCGCCGCT) in exon 8 were deleted. For *rbfox2*, 2 bp (TCAATGAAAGGGGC) in exon 3 were deleted. For *rbfox1l*, the PCR primers: 5ʹ-GCAGTTTGTCGTTTTGCCCA-3ʹ and 5ʹ- ACTGACCCTGGGTAGGCTG-3ʹ were used for genotyping to produce a 193 bp wildtype amplicon and a 179 bp mutant (*chb5*) amplicon. For *rbfox2*, the PCR primers: 5ʹ-TTGGAAAGATTCTGGATGTG-3ʹ and 5ʹ- TGACGAGTTTTGGAGGTTGA-3ʹ were used to produce a 189 bp wildtype amplicon and a 187 bp mutant (*chb6*) amplicon.

### Construction of the *Tg(myl7:GFP-rbfox1l)* strain

A construct containing the following DNA elements was assembled by Gibson cloning: (1) a ~0.9 kb *myl7* promoter to drive specific expression in cardiomyocytes; (2) a bicistronic cassette encoding an enhanced green fluorescent protein (GFP) and zebrafish Rbfox1l, separated by a viral P2A sequence and 3xHA tag. The entire construct was flanked with Tol2 sites to facilitate transgenesis. In this line, all cardiomyocytes constitutively express GFP and Rbfox1l. The full name of this line is *Tg(myl7:GFP-P2A-3xHA-rbfox1l)*^*chb7*^.

### Microscopy, imaging, and functional analysis

For compound microscopy, zebrafish embryos were anesthetized in 0.16% Tricaine (Sigma-Aldrich), immobilized in 0.9% low-melt agarose dissolved in embryo media (E3 medium) on depression slides, and imaged using epifluorescence and/or bright-field optics on a Nikon Eclipse 80i compound microscope with a QImaging Retiga 2000R CCD camera (QImaging).

For live imaging of the beating heart over successive cardiac cycles, 72 and 30 hpf zebrafish embryos carrying the *Tg(myl7:GFP)* myocardial reporter were anesthetized in 0.16% Tricaine (Sigma–Aldrich), immobilized on a depression slide in 0.9% low melt agarose dissolved in embryo media (E3 medium), and imaged on a Nikon Eclipse 80i compound microscope for 15 s with a QImaging Retiga 2000R CCD camera (QImaging). For live imaging on a confocal microscope, embryos carrying fluorescent reporters were anesthetized in 0.16% Tricaine and mounted on 35 mm glass bottom Petri dishes (MatTek Corp) in 0.9% low melting point agarose containing 0.16% Tricaine and covered in embryo media with 0.4% Tricaine. For imaging fixed embryos on a confocal microscope, animals were embedded in 0.9% low melting point agarose on 35 mm glass bottom Petri dishes (MatTek Corp). Z-stack images were acquired using an Olympus FV3000R resonant scanning confocal microscope and processed using Fiji software^41^.

For functional analysis at 72 hpf, still images of each chamber at the end of diastole and systole were extracted from movies and area measurements made using Fiji software^41^. To calculate percent fractional area change, the following formula was used: (end diastolic area − end systolic area) / end-diastolic area × 100. To quantify function at 30 hpf, still frames of the arterial pole, which was defined as the first third of the linear heart tube, were extracted during diastole and systole and the width measured using Fiji software^41^. To calculate percent fractional shortening, the same formula was used where width was substituted for area. To quantify chamber volumes at 48 hpf, hearts from live embryos were imaged over successive cardiac cycles using a Zeiss Lightsheet Z.1 microscope. Chamber volumes at the end of diastole and systole were determined in MATLAB using the CFIN deep learning platform and used to calculate ejection fraction, stroke volume, and cardiac output^19^.

Cardiomyocyte area and circularity measurements were made on *Tg(myl7:GFP*) embryos stained with anti-GFP and anti-ALCAMA (ZN-8) antibodies using Fiji software^42^.

### Whole mount immunofluorescence

Embryos were anesthetized with 0.16% Tricaine prior to overnight fixation in phosphate-buffered saline (PBS) containing 4% paraformaldehyde (PFA). The following day, embryos were rinsed and washed in PBS containing 0.1% Tween-20 (PBST) to remove PFA and treated with bleaching solution to remove pigment (0.8% KOH, 0.9% H_2_O_2_, and 0.1% Tween-20 in ddH_2_O). Next, embryos were washed with PBST and permeabilized with PBS containing 0.5% Triton X-100 for 2 h. Embryos were treated with blocking buffer (5% bovine serum albumin (BSA) and 5% goat serum in PBST) for 1 h prior to overnight incubation at 4 °C with primary antibodies diluted in blocking buffer. The following primary antibodies were used: anti-GFP (1:500 dilution, Abcam, ab13970), anti-mCherry (1:200, Abcam, ab125096), anti-Rbfox1l (1:200)^16^, anti-Rbfox2 (1:200)^16^, anti-Myosin Heavy Chain (1:50, MF20, Developmental Studies Hybridoma Bank (DSHB)), anti-Eln2 (1:1000)^43^, anti-ALCAMA (1:50, ZN-8, DSHB), anti-Atrial Myosin Heavy Chain (1:50, S46, DSHB), anti-Tropomyosin (1:200, CH1, DSHB), and anti-Troponin T (1:500, CT3, DSHB). Embryos were washed in PBST and incubated with Alexa Fluor-conjugated secondary antibodies (1:500, Thermo Fisher Scientific) for 2 h at room temperature. The following secondary antibodies were used: Goat anti Rabbit IgG 488 (A11008), Goat anti Mouse IgG2a 488 (A21131), Goat anti Mouse IgG2b 568 (A21144), Goat anti Rabbit IgG 568 (A11036), Goat anti Mouse IgG1 568 (A21124), Goat anti Mouse IgG2a 555 (A-21137), Goat anti Chicken IgY 488 (A11039), Goat anti Rabbit IgG 647 (A21244). Following incubation, embryos were washed in PBST and nuclei labeled with DAPI for 5 min (1:1000, Sigma–Aldrich) for imaging.

### EdU proliferation analysis

EdU (5-ethynyl-2ʹ-deoxyuridine) labelling was preformed using the Click-iT™ Plus EdU Cell Proliferation Kit (Thermo Fisher Scientific, C10640). Briefly, embryos were treated with 1 mM EdU in embryo media (E3 medium) for 24 h followed by fixation and processing according to the immunofluorescence protocol with the following modifications. After bleaching and permeabilization, embryos were incubated in Click-iT® Plus reaction cocktail for 1 h in the dark, followed by several washes with PBST. Thereafter, embryos were incubated in blocking buffer, primary antibodies and secondary antibodies in the dark according to the immunofluorescence protocol.

### Kaede photoconversion

*Tg(nkx2.5:Kaede)*^*fb9*^ embryos were photoconverted by exposing Kaede protein that was localized in pharyngeal arch 2 (PA2) to fluorescent light passed through the DAPI filter on an Olympus FV3000R resonant scanning confocal microscope with a 30×objective. At 28 h post fertilization (hpf), embryos were mounted in 0.9% low-melt agarose in a 35 mm glass-bottom dish (MatTek Corp.). Prior to photoconversion, embryos were imaged using a GFP filter. Immediately thereafter, PA2 was exposed continually to DAPI light for 10 s and assessed visually for residual green fluorescence. As needed, embryos were exposed to DAPI light for an additional 5 s, which was generally sufficient for complete photoconversion. Embryos were immediately imaged using the GFP and RFP filters, removed from agarose, arrayed individually in six-well plates, and incubated in the dark until 72 hpf when they were imaged again by confocal microscopy. During imaging, embryos were anesthetized in 0.16% Tricaine to cease cardiac contractions.

### Isolation of hearts and RNA for RNA-seq analysis

Embryos carrying *myl7:GFP* reporter were anesthetized with 0.16% Tricaine at 48 hpf and their hearts dissected in ice-cold PBS under a Leica Mz16 F Fluorescence Stereo Microscope. Pooled hearts (*n* = 35–70) were lysed in TRIzol reagent (Invitrogen) and total RNA purified by RNA microPrep (Zymo Research) for each biological replicate. The purified RNA eluates were kept at −80 °C prior to sequencing or qPCR. Total RNA from 16 pools (8 biological replicates of control and *rbfox*-null) were analyzed by TapeStation (Agilent). RNA was processed with poly(A) selection for full length transcripts and libraries generated using the ultra-low input library preparation kit (Illumina). Libraries were sequenced on an Illumina HiSeq platform using the 2 x 150 bp configuration with single index.

### RNA-seq data processing and splicing analysis

RNA-seq mapping and quantitation was done using STAR v. 2.6.1a^44^ with flags --runMode alignReads mThreadN 10 --genomeDir GenomeDir --outSAMtype BAM Unsorted --quantMode TranscriptomeSAM --outSAMstrandField intronMotif with --genomeDir pointing to a GRCz11/danRer11 STAR index. The mapped reads were further analyzed by HTSeq-count v.0.11.2^45^ and annotated using RefSeq database^46^. The expression levels for each transcript were quantified by FPKM (Fragments Per Kilobase of transcript per Million mapped reads). For genes with multiple isoforms, the FPKM values were summed across all isoforms as the expression values for the genes. Differential expression analysis was performed using the default DESeq2 v.1.32.0 method^47^ on count data comparing DKO (*rbfox1l*^*−/−*^; *rbfox2*^*−/−*^) vs. sibling control (CTRL) samples. P-values obtained by the Wald test (two-sided) were corrected for multiple testing using the Benjamini and Hochberg method. Genes with Benjamini-Hochberg adjusted *p*-values less than 0.05 and more than 1.5-fold change were considered differentially expressed. Only genes with FPKM greater than 5 in at least one sample were kept for further analysis. Gene ontology analysis was performed using DAVID v.6.8^48,49^. P-values obtained by a modified Fisher’s exact test (EASEscore) were corrected for multiple testing using the Benjamini and Hochberg method.

RNA-seq mapping and quantitation was done against GRCz11/danRer11 annotation using STAR v. 2.6.1a as described above. The dedicated splicing analysis algorithm rMATS v.4.2^23^ was run on the data as rmats.py with flags --readLength 150 --nthread -t paired pointing to the same GTF file and STAR index. Event inclusion was quantified both by counting junction reads only, and both junction and read on targets. The resulting data collated across replicates were filtered, and events had to meet the following conditions to be retained: the event had to be called in all samples, differences in inclusion levels had to be ≥0.2 or ≤ −0.2 and false-discovery rate < 0.05. *P*-values were obtained by a likelihood-ratio test and the false discovery rate (FDR) was based on the Benjamini-Hochberg approach.

### Quantitative and semi-quantitative RT-PCR

Embryo hearts were pooled and lysed in TRIzol reagent (Invitrogen) and total RNA purified by RNA Direct-zol Microprep (Zymo Research) for each biological replicate as described above. Reverse transcription of cDNA was synthesized using the Superscript III first-Strand Synthesis System (Invitrogen). Real-time quantitative RT-PCR was performed in triplicate with the Quant Studio3 Real-Time PCR System (Thermo Fisher Scientific) with Fast SYBR Green PCR Master Mix (Thermo Fisher Scientific). The 2^−ΔΔCT^ method^50^ was used to measure differential expression levels after normalization to *rsp11*. Isoform specific quantitative RT-PCR was performed using at least one primer specific to the alternatively spliced exon as determined by rMATS. Total transcript levels were determined using primer pairs that bind to regions conserved across all isoforms. Semi-quantitative RT-PCR was performed using Phusion polymerase (Thermo Scientific) to amplify the cassette exon and flanking sequences. Products were run on 4% agarose gels and imaged with the BioRad Gel Doc XR + imager. Band intensities were quantified with Fiji and normalized to background signal prior to calculation of PSI. All primer sequences used for RT-PCR are shown in Supplementary Table 5.

### Transmission electron microscopy (TEM)

Embryos were fixed in a 1:1 mixture of embryo media (E3 medium) and FGP fixative (2.5% paraformaldehyde (PFA), 5% glutaraldehyde, and 0.06% picric acid in 0.2 M cacodylate buffer) overnight. Fixed embryos were washed in 0.1 M cacodylate buffer several times and osmicated in 1% Osmium tetroxide/1.5% Potassium ferrocyanide in ddH_2_O for 3 h followed by several washes of in ddH_2_O. Embryos were stained with 1% uranyl acetate in maleate buffer (pH 5.2) for 1 h. After washing several times in malelate buffer, embryos were dehydrated by a graded cold ethanol series that was changed three times over one hour to reach 100%. Embryos were immersed in propylene oxide with two solution changes over one hour. Embryos were placed in a 1:1 mixture of propylene oxide and Taab 812 resin (Marivac-Canemo Inc.) including catalyst overnight at 4 °C. The next day, embryos were embedded in pure Taab resin mixture and placed in a 60 °C incubator for 48 h. Samples containing the heart region were cut as 80 nm sections using a Leica Ultracut S microtome, mounted on formvar-carbon coated slot Cu grids, and stained with 0.2% Lead Citrate. Cardiomyocytes in the ventricle were viewed and imaged under a Philips Tecnai BioTwin Spirit or JEOL 1200x Electron Microscope.

### Measurement of in vivo oxygen consumption rates (OCR)

Embryos produced from pairwise matings were de-chorionated manually at 24 hpf and assayed at 48 hpf in an Agilent Seahorse XFe96 Analyzer. One embryo was added per well to a Seahorse XFe96 Spheroid Microplates (Agilent, 102978-100; FluxPak: Agilent, 102601-100) in a total volume of 180 μl of E3 containing 0.16% Tricaine using a cut P1000 pipet tip to avoid air bubbles. Oligomycin (Sigma–Aldrich, 75351-5MG) was loaded into port A at a final concentration of 25 μM. Carbonyl cyanide 4-(trifluoromethoxy) phenylhydrazone (FCCP; Sigma–Aldrich, C2920-10MG), was loaded into port B at a final concentration of 2.5 μM. Rotenone (Sigma–Aldrich, R8875-1G) and antimycin (Sigma–Aldrich, A8674-25MG) were loaded into port C at final concentrations of 2 μM. The running program consisted of 24 measurement cycles, including 6 cycles for baseline, and 6 cycles after treatment with each drug. Each cycle includes 2 min mix, 1 min wait and 2 min measure. The temperature of the Analyzer was set to 28 °C, which was maintained by desk fans and dry ice to circulate the cool air. Oxygen consumption rate (OCR) values were determined using the Seahorse software, Wave 2.4.0. GraphPad Prism 9 software was used for statistical analysis.

### Metabolite profiling and mass spectrometry

For sample preparation, fifty embryos were dechorionated using pronase (0.5 mg/ml, Sigma) and transferred to a 1.5-ml tube. To dissolve the yolk, embryos were pipetted up and down with a P1000 in 1000 ul of cold Ringer’s working solution with phosphatase inhibitor (Roche, 04906845001) and protease inhibitor (Roche, 11873580001) according to *The Zebrafish Book*^51^. Deyolked embryos were obtained by spinning at 4 °C for 2 min at 13,800 × *g* and removing the supernatant. 60% LC-MS Methanol in LC-MS Chloroform was added to the embryo pellet to extract the metabolites and dried down using the Savant SPD111V SpeedVac Concentrator. The sample extract was separated using an iHILIC-(P) Classic column (5 μm, 150 × 2.1 mm I.D., HILICON) coupled to a Thermo Scientific SII UPLC system. The sample extracts were run using Buffer A: water with 20 mM ammonium carbonate with 0.1% ammonium hydroxide and Buffer B: acetonitrile. The gradient was run at a flow rate of 0.150 mL/min as follows: 0–20 min linear gradient from 80% to 20% B; 20–20.5 min linear gradient from 20% to 8% B; 20.5–28 min hold at 80% B; 28–30 min hold to waste at 80% B. Mass spectrometry detection was carried out on a Q Exactive HF-X orbitrap mass spectrometer with a HESI source operated in negative ion mode with a full-scan analysis over 70–1000 m/z and high resolution (60,000) was used for mass detection. Sigma’s MSMLS (Mass Spectrometry Metabolite Library) was run in both negative and positive ion modes. A total of 537 compounds were detected, of which 286 metabolites were detected in negative ion mode. This negative ion mode library was subsequently used for metabolite identification. A master mix of metabolic reference standards were run prior to each set of samples, such that there was no retention time shift within the margins of 5 ppm. Sample QC/.data processing were completed using the Thermo Tracefinder 3.3 analysis software (Thermo Fisher Scientific) for peak detection and integration. The metabolic peak area was first normalized to the internal standard (glutarate 1 ug/sample) and then to the protein concentration to obtain the final intensity value(s) for which the metabolite levels were normalized to the total protein amount (μg) per sample. The protein layer was also dried down in the Savant SPD111V SpeedVac Concentrator and protein was extracted from the pellets in 50 ul of 0.2 M sodium hydroxide by using a heat block set to 95 °C for 20 min. The protein concentration was then read on the BioTek Epoch Microplate Spectrophotometer.

### Determination of NAD+/NADH ratio

NAD+/NADH measurements were perform using NAD/NADH-Glo Assay (Promega, G9071) according to manufacturer’s instructions with some modification. For sample preparation, three embryos were pooled in 50 μl PBS, homogenized and lysed in 50 μl of 0.2 N sodium hydroxide with 1% dodecyltrimethylammonium bromide (DTAB, Sigma, D8638) using a cordless pellet pestle (Sigma, Z359971). To measure NAD+, 40 μl of each sample was transferred to a PCR tube containing 20 μl of 0.4 N hydrogen chloride (HCl) and heated to 60 °C to selectively degrade NADH. Samples were equilibrated for 10 min at room temperature and neutralized by adding 20 μl of 0.5 M Trizma base. To measure NADH, 40 μl of each sample was transferred to a PCR tube and incubated at 60 °C for 15 min to degrade NAD+. After 10 min equilibration at room temperature, 40 μl of 0.25 M Trizma/HCl solution (0.25 M Trizma base in 0.2 N HCl) was added to neutralize the base-treated samples. For the NAD/NADH-Glo Assay, 25 μl of the above samples were added to 25 μl of the NAD/NADH-Glo Detection Reagent in a 96-well plate and luminescence was recorded by FlexStation 3 Multi-Mode Microplate Reader (Molecular Devices, LLC.) every 5 min for 1.5 h.

### Microinjection with wildtype and mutant human *RBFOX2* mRNA

To generate wildtype human *RBFOX2* mRNA, full length *RBFOX2* (Addgene, Plasmid #59770) was released from the Addgene plasmid with StuI and XhoI and subcloned into the pCS2+ vector. For mutant *RBFOX2* mRNA, three mutant cDNAs were created based on the variants that segregate with HLHS in newborns^11^. Variant 1 is a frameshift mutation of A899AG at position 36157314 of chromosome 22; Variant 2 is a nonsense mutation of G1072A at position 36155975; Variant 3 is splice site mutation of G1312C at position 36141969. These variants were subcloned into pCS2+. Plasmids were linearized with SacII and mRNA synthesized using the mMessage mMachine SP6 transcription kit (Thermo Fischer Scientific). Embryos were injected at the one-cell stage with ∼1 nl (100 pg) of full-length wildtype or mutant *RBFOX2* mRNA.

### Statistics

Data are expressed as the mean ± SD of at least three independent experiments. Statistical significance was evaluated by unpaired two-tailed Student’s *t*-tests in GraphPad Prism 9. *P* < 0.05 was considered significant. All sample sizes are reported in the figure legends.

### Reporting summary

Further information on research design is available in the Nature Research Reporting Summary linked to this article.

## Supplementary information

Supplementary Information

Reporting Summary

Description of Additional Supplementary Files

Supplementary Dataset 1

Supplementary Dataset 2

Supplementary Dataset 3

Supplementary Dataset 4

Supplementary Dataset 5

Supplementary Movie 1

Supplementary Movie 2

## Data Availability

The data supporting the findings from this study are available within the manuscript and its Supplementary Information. RNA-Seq raw data have been deposited in the GEO under accession number GSE189934. Metabolomics data have been deposited in Metabolights^52^ under the unique identifier MTBLS4176. Source data are provided with this paper.

## Source data

Source Data
