# Supplementary material for: Intrinsic myocardial defects underlie an Rbfox-deficient zebrafish model of hypoplastic left heart syndrome
Source: Nat Commun. 2022 Oct 5;13:5877. doi: 10.1038/s41467-022-32982-x (PMC9534849; doi:10.1038/s41467-022-32982-x)
Supplement: Supplementary file 1 — Supplementary Information [file 41467_2022_32982_MOESM1_ESM.pdf]

Intrinsic myocardial defects underlie an Rbfox deficient zebrafish model of hypoplastic left heart syndrome

Mengmeng Huang[1,2,^], Alexander A. Akerberg[1,2,^], Xiaoran Zhang[1,2], Haejin Yoon[3], Shakchhi Joshi[3], Celia Harding[1], Christopher Nguyen[2,4,5], William T. Pu[1,2,6], Marcia C. Haigis[3], C. Geoffrey Burns[1,2,*], Caroline E. Burns[1,2,6,*]

Supplementary Information

Supplementary Figures 1-4

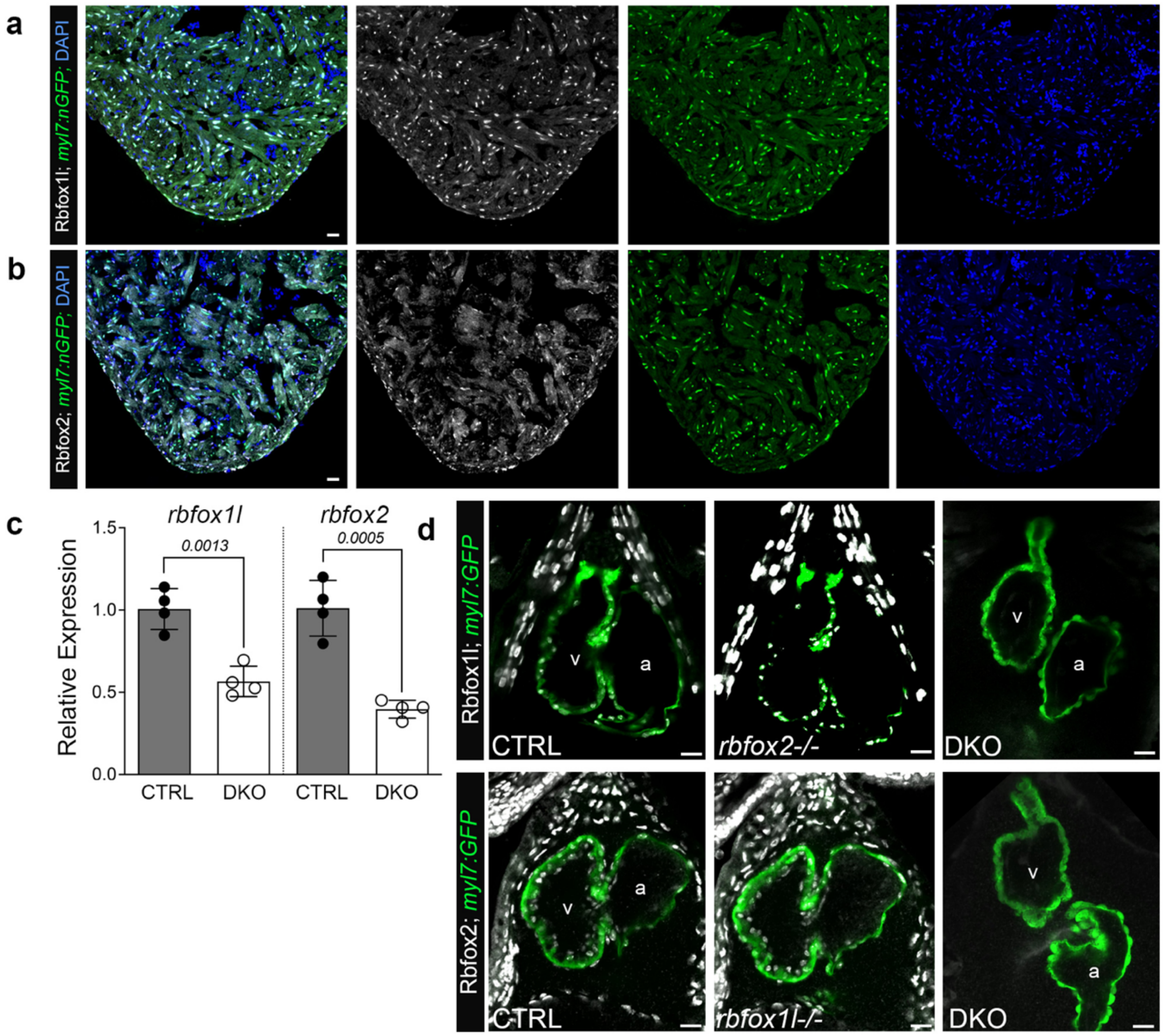

Supplementary Fig 1 – Expression of Rbfox1l and Rbfox2 in adult zebrafish ventricles and validation of the Rbfox antibodies and *rbfox1l and rbfox2* null alleles. **a,b** Confocal image of ventricular section from adult zebrafish carrying the *Tg(myl7:nucGFP)* transgene co-immunostained with antibodies that detect Rbfox1l (white; a), Rbfox2 (white; b), and GFP (green), and counterstained with DAPI (blue). Single and merged channel images are shown. **c** Bar graph showing the relative expression levels of *rbfox1l* and *rbfox2* transcripts in 72 hpf CTRL and DKO embryos measured by real-time quantitative RT-PCR. **d** Confocal section of hearts in 72 hpf CTRL, *rbfox2*$^{-/-}$ or *rbfox1l*$^{-/-}$ single mutants, or DKO zebrafish embryos carrying the *Tg(myl7:GFP*) reporter co-immunostained with antibodies that detect Rbfox1l (white) or Rbfox2 (white) and GFP (green) as labeled. **Sample sizes and statistics:** (a,b) Little to no variation in adult cardiac expression was observed between animals in each group (n=6 per group). (c) For real-time quantitative RT-PCR, n=4 biological replicates per cohort were analyzed with 15 whole embryos/replicate. Three technical replicates were run for each biological replicate. Data are presented as mean values +/- one SD. Statistical significance was determined by an unpaired, two-tailed Student's t-test assuming equal variances. P values are shown. Source data are provided as a Source Data file. (d) Little to no variation in cardiac expression was observed where n=15 embryos were analyzed per group. Abbreviations: v, ventricle; a, atrium. Scale bars: 20 μm.

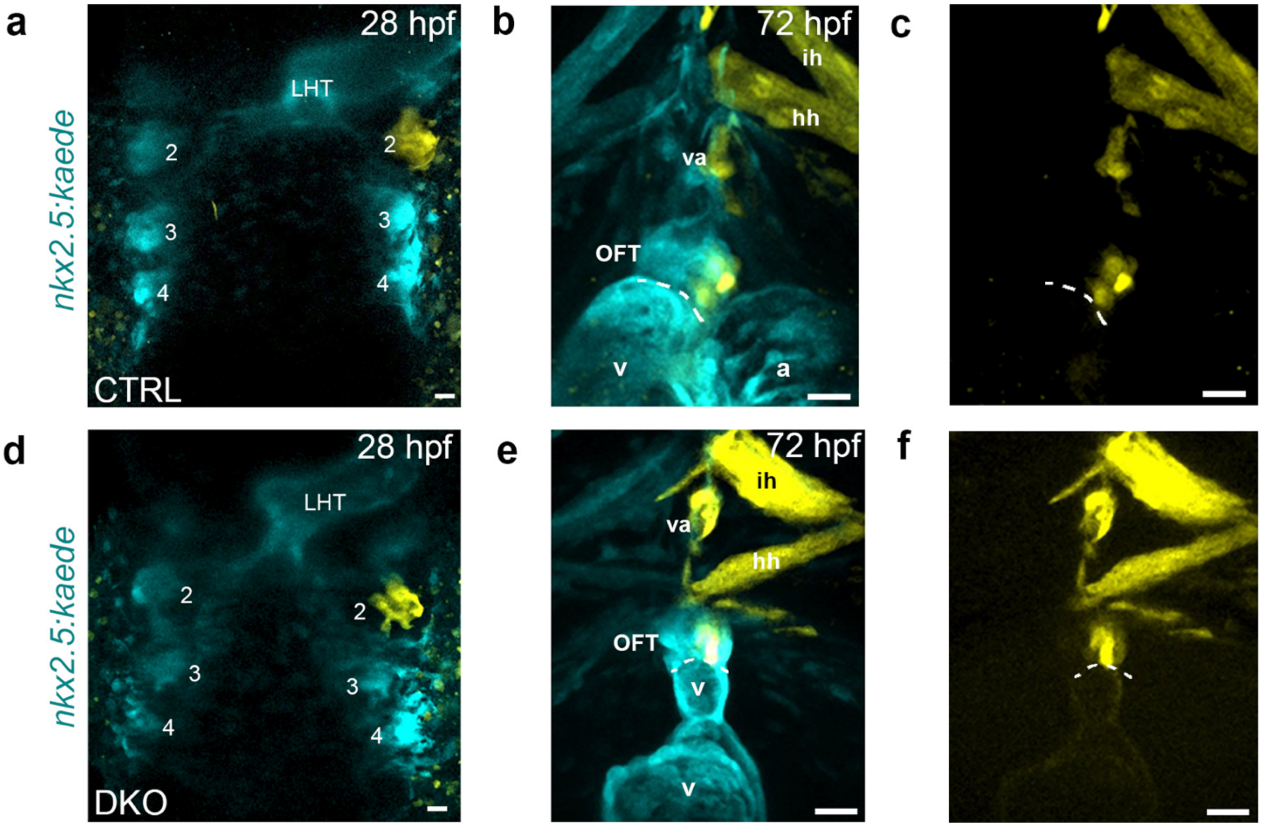

Supplementary Fig 2– Lineage tracing to distinguish ventricular from OFT myocardium. a,d Merged confocal z-stacks of 28 hpf CTRL and DKO embryos carrying the *Tg(nkx2.5:Kaede)* reporter (cyan) immediately following unilateral photoconversion of Kaede in pharyngeal arch 2 (PA2, yellow). Numbers label the pharyngeal arches. Dorsal view, anterior up. **b,c,e,f** Confocal z-stacks of the same embryos in a and d at 72 hpf shown as merged (b,e) or single-channel (c,f) images. Dotted white lines in c and f demarcate the boundary between the ventricle and OFT based on the yellow signal from the lineage trace. Ventral views, anterior up. **Sample sizes and statistics:** Little to no variation in expression pattern was observed between animals in each group (n=10/cohort). Abbreviations: LHT, linear heart tube; OFT, outflow tract; v, ventricle; a, atrium; va, ventral aorta; ih, interhyal; hh, hyohyal. Scale bars: 20 µm.

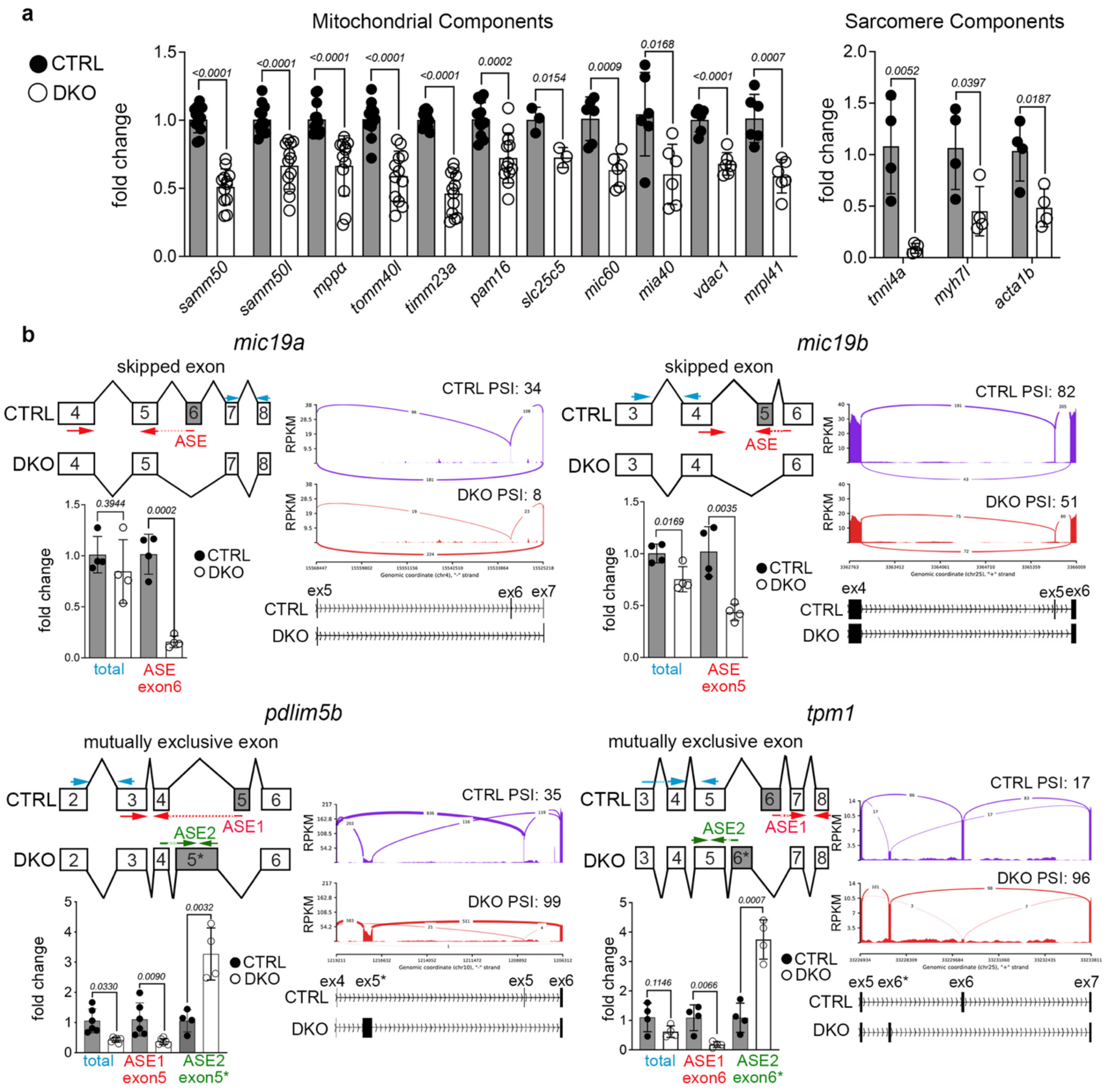

Supplementary Fig 3 – Validation of differential expression and alternative splicing events by real-time quantitative RT-PCR. a Graph showing quantitative real-time RT-PCR validation of differential gene expression detected by RNAseq between hearts isolated from CTRL and DKO embryos at 48 hpf. **b** Schematics of 4 alternatively spliced transcripts with primer locations shown to detect total transcript levels (blue) or isoforms with alternatively spliced exons (ASE transcript levels; red or green) by quantitative real-time RT-PCR. Graphs show normalized fold change in total or ASE transcript levels between hearts isolated from CTRL and DKO embryos. Shashimi plots derived from rMATS data are shown for each ASE with associated percent spliced in (PSI) values. **Sample sizes and Statistics:** (a,b) For real-time quantitative RT-PCR, n=12 biological replicates per cohort for *samm50, samm50l, mppa, tomm20l, timm23a*, and *pam16*, n=3 biological replicates for *slc25c5*, n=6 biological replicates

for *mic60, mia40, vdac1*, and *mrpl41*, n=4 biological replicates for *tnni4a, myh7l, acta1b, mic19a* (total and ASE exon6), *mic19b* (total and ASE exon5), n=6 biological replicates for *pdlim5b* (total and ASE1 exon5), n=4 biological replicates for *pdlim5b* (ASE2 exon5*), *tpm1* (total, ASE1 exon6, and ASE2 exon6*). Each biological replicate consisted of ~35 pooled hearts and each dot on the graph represents one biological replicate. Three technical replicates were run for each biological replicate. Data are presented as mean values +/- one SD. Statistical significance was determined by an unpaired, two-tailed Student's t-test assuming equal variances. P values are shown. Source data are provided as a Source Data file.

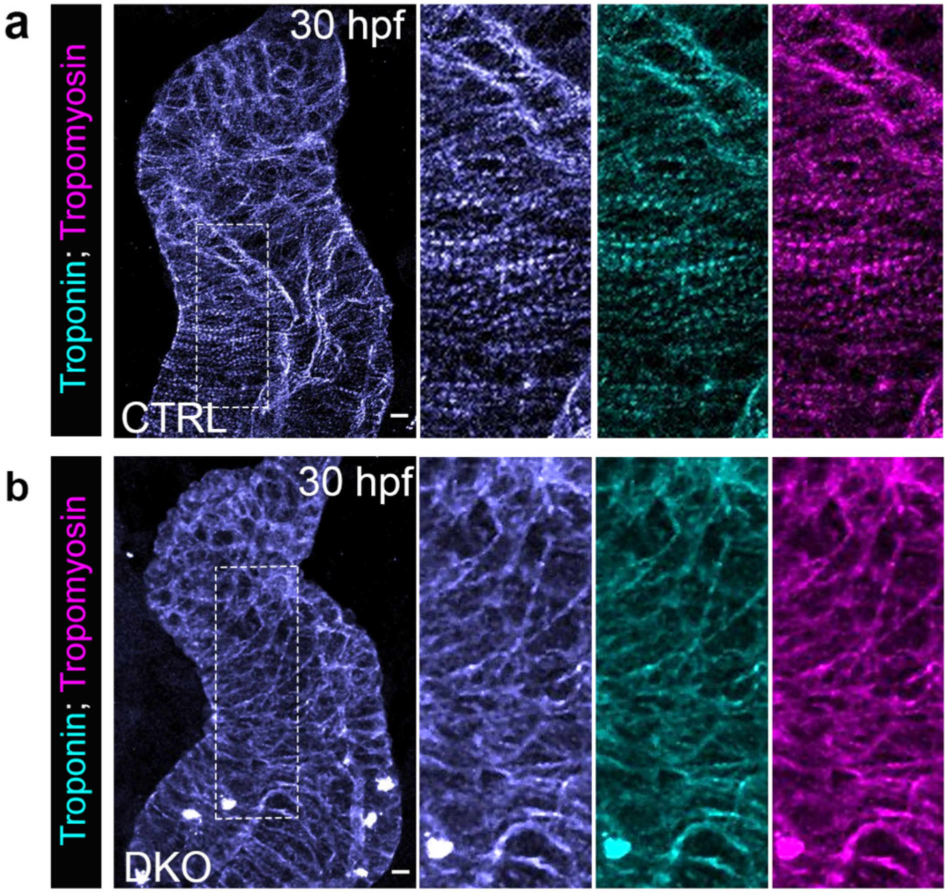

Supplementary Fig 4 – Rbfox proteins are essential for myofibrillar organization at linear heart tube stages in zebrafish. a,b Confocal projections of 30 hpf linear heart tubes in CTRL and DKO embryos immunostained with anti-Troponin T (cyan) and anti-Tropomyosin (magenta) to visualize thin filaments. Boxed regions are shown in higher magnification views as merged and split channels. **Sample sizes and statistics:** Little to no variation in myofibrillar organization was observed between animals in each group (n=12 embryos/group). Scale bars: 20 µm.
