# Supplementary material for: Intrinsic myocardial defects underlie an Rbfox-deficient zebrafish model of hypoplastic left heart syndrome
Source: Nat Commun. 2022 Oct 5;13:5877. doi: 10.1038/s41467-022-32982-x (PMC9534849; doi:10.1038/s41467-022-32982-x)
Supplement: Supplementary file 2 — Reporting Summary [file 41467_2022_32982_MOESM2_ESM.pdf]

nature portfolio

Corresponding author(s): C. Geoffrey Burns, Caroline E. Burns

Last updated by author(s): 2022/07/06

Reporting Summary

Nature Portfolio wishes to improve the reproducibility of the work that we publish. This form provides structure for consistency and transparency in reporting. For further information on Nature Portfolio policies, see our Editorial Policies and the Editorial Policy Checklist.

Statistics

For all statistical analyses, confirm that the following items are present in the figure legend, table legend, main text, or Methods section.

n/a	Confirmed	
☐	☒	The exact sample size (*n*) for each experimental group/condition, given as a discrete number and unit of measurement
☐	☒	A statement on whether measurements were taken from distinct samples or whether the same sample was measured repeatedly
☐	☒	The statistical test(s) used AND whether they are one- or two-sided<br>*Only common tests should be described solely by name; describe more complex techniques in the Methods section.*
☒	☐	A description of all covariates tested
☐	☒	A description of any assumptions or corrections, such as tests of normality and adjustment for multiple comparisons
☐	☒	A full description of the statistical parameters including central tendency (e.g. means) or other basic estimates (e.g. regression coefficient) AND variation (e.g. standard deviation) or associated estimates of uncertainty (e.g. confidence intervals)
☐	☒	For null hypothesis testing, the test statistic (e.g. *F*, *t*, *r*) with confidence intervals, effect sizes, degrees of freedom and *P* value noted<br>*Give P values as exact values whenever suitable.*
☒	☐	For Bayesian analysis, information on the choice of priors and Markov chain Monte Carlo settings
☒	☐	For hierarchical and complex designs, identification of the appropriate level for tests and full reporting of outcomes
☒	☐	Estimates of effect sizes (e.g. Cohen's *d*, Pearson's *r*), indicating how they were calculated

Our web collection on statistics for biologists contains articles on many of the points above.

Software and code

Policy information about availability of computer code

Data collection	Compound microscopy images were acquired with NIS Elements AR v.4.30.02. Confocal microscopy images were acquired using Fluoview FV31S-Sw v.2.4.1.198. qRT-PCR data was acquired using Quant Studio Design and Analysis Software v.1.5.1. LSFM images were acquired with Zeiss Zen 2019.
Data analysis	Volumetric measurements of zebrafish heart function were made using CFIN as described by Akerberg et al. (PMC6826023). CFIN was run using MATLAB v.2018a (MathWorks). RNA-seq mapping and quantitation was done using STAR v. 2.6.1a. The mapped reads were further analyzed by HTSeq-count v.0.11.2. Differential expression analysis was performed using DESeq2 v.1.32.0. Gene ontology analysis was performed using DAVID v.6.8. The dedicated splicing analysis algorithm rMATS v.4.2 was used to determine alternative splicing events. Seahorse data was analyzed using Wave 2.4.0. GraphPad Prism v.9.3.1 was used for statistical analysis. Fiji (Image J v.2.3.0/1.53f) was used for semiquantitative RT-PCR analysis and for image processing. Mass spectrometry QC/data analysis was performed using Thermo Tracefinder 3.3. Adobe Photoshop v. 23.4.1 was used for image processing and figure generation.

For manuscripts utilizing custom algorithms or software that are central to the research but not yet described in published literature, software must be made available to editors and reviewers. We strongly encourage code deposition in a community repository (e.g. GitHub). See the Nature Portfolio guidelines for submitting code & software for further information.

Data

Policy information about availability of data

All manuscripts must include a data availability statement. This statement should provide the following information, where applicable:

- Accession codes, unique identifiers, or web links for publicly available datasets
- A description of any restrictions on data availability
- For clinical datasets or third party data, please ensure that the statement adheres to our policy

RNA-sequencing datasets summarized in tables S1-S3, were deposited into the NCBI GEO repository under accession number GSE189934. To review, go to https://www.ncbi.nlm.nih.gov/geo/query/acc.cgi?acc=GSE189934

Metabolomics data summarized in Supplementary Table 4 were deposited into the Metabolights repository under the accession number MTBLS4176. To review, go to https://www.ebi.ac.uk/metabolights/MTBLS4176/descriptors

Field-specific reporting

Please select the one below that is the best fit for your research. If you are not sure, read the appropriate sections before making your selection.

☒ Life sciences ☐ Behavioural & social sciences ☐ Ecological, evolutionary & environmental sciences

For a reference copy of the document with all sections, see nature.com/documents/nr-reporting-summary-flat.pdf

Life sciences study design

All studies must disclose on these points even when the disclosure is negative.

Sample size	Sample sizes were chosen based on accepted standards in the field, previously published literature, and our experience in observing statistically significant differences of similar effects in zebrafish embryos: Mahmoud AI et al. 2015; PMID: 26256209; Mosimann C et al. 2015; PMID 26306682; Akerberg A et al. 2017; PMID 28372944; Abrial M et al. 2022; PMID 35098309.
Data exclusions	No data were excluded from the analyses.
Replication	All experimental results were verified with at least 3 biological replicates. Each biological replicate was the product of multiple distinct animal crosses that were performed independently. All qPCR and RNA-seq replicates consisted of material from pooled hearts or embryos.
Randomization	Rbfox2/1l DKO zebrafish embryos were isolated based on genotype and compared to siblings from the same clutch. Zebrafish embryos used in this study were collected at stages that precede sexual dimorphism and as such, embryos within each group (sibling controls and DKO) were indistinguishable from each other.
Blinding	Assays pertaining to rbfox2 DKO vs CTRL phenotypes were inherently blind in that the respective genotypes were not determined until after data was collected and analyzed. All other assays such as quantitative RT-PCR, CFIN, and metabolomics (Seahorse) assays were conducted using automated computational programs.

Reporting for specific materials, systems and methods

We require information from authors about some types of materials, experimental systems and methods used in many studies. Here, indicate whether each material, system or method listed is relevant to your study. If you are not sure if a list item applies to your research, read the appropriate section before selecting a response.

Materials & experimental systems

n/a	Involved in the study	
☐	☒	Antibodies
☒	☐	Eukaryotic cell lines
☒	☐	Palaeontology and archaeology
☐	☒	Animals and other organisms
☒	☐	Human research participants
☒	☐	Clinical data
☒	☐	Dual use research of concern

Methods

n/a	Involved in the study	
☒	☐	ChIP-seq
☒	☐	Flow cytometry
☒	☐	MRI-based neuroimaging

Antibodies

Antibodies used	The following primary antibodies were used: anti-GFP (Abcam, ab13970), anti-mCherry (Abcam, ab125096), anti-Rbfox1l (Berberoglu et al., 2017; supplied by the Amacher lab (Ohio State University)), anti-Rbfox2 (Berberoglu et al., 2017; supplied by the Amacher lab

(Ohio State University)), anti-Myosin Heavy Chain (MF20, Developmental Studies Hybridoma Bank (DSHB)), anti-Eln2 (Miao et al., 2007; gifted by the Keeley lab (University of Toronto)), anti-ALCAMA (ZN-8, DSHB), anti-Atrial Myosin Heavy Chain (S46, DSHB), anti-Tropomyosin (CH1, DSHB), and anti-Troponin T (CT3, DSHB). Secondary antibodies (Thermo Fisher) : Goat anti Rabbit IgG 488 (A11008), Goat anti Mouse IgG2a 488 (A21131), Goat anti Mouse IgG2b 568 (A21144), Goat anti Rabbit IgG 568 (A11036), Goat anti Mouse IgG1 568 (A21124), Goat anti Mouse IgG2a 555 (A-21137), Goat anti Chicken IgY 488 (A11039), Goat anti Rabbit IgG 647 (A21244).

Validation

anti-GFP (Abcam, ab13970), https://www.abcam.com/gfp-antibody-ab13970.html
anti-mCherry (Abcam, ab125096), https://www.abcam.com/mcherry-antibody-1c51-ab125096.html
anti-Rbfox1l, anti-Rbfox2 (Berberoglu et al., 2017), https://www.ncbi.nlm.nih.gov/pmc/articles/PMC5437870/
anti-Eln2 (Miao et al., 2007), https://pubmed.ncbi.nlm.nih.gov/17112714/
anti-Myosin Heavy Chain (MF20, DSHB), https://dshb.biology.uiowa.edu/MF-20
anti-ALCAMA (ZN-8, DSHB), https://dshb.biology.uiowa.edu/ZN-8
anti-Atrial Myosin Heavy Chain (S46, DSHB), https://dshb.biology.uiowa.edu/S46
anti-Tropomyosin (CH1, DSHB), https://dshb.biology.uiowa.edu/CH1
anti-Troponin T (CT3, DSHB), https://dshb.biology.uiowa.edu/CT3

Animals and other organisms

Policy information about studies involving animals; ARRIVE guidelines recommended for reporting animal research

Laboratory animals

We used zebrafish (Danio rerio) embryos (24-72 hours post fertilization) and both female and male adults (3-month to 2-year old). The following zebrafish strains were used in this study: Tg(myl7:GFP)f1 (Burns et al., 2005), Tg(myl7:nucGFP)fb18 (González-Rosa et al., 2018), Tg(kdrl:mCherry)s896 (Chi et al., 2008), TgBAC(nkx2.5:Kaede)fb9 (Paffett-Lugassy et al., 2013) , rbfox1lchb5 (this study), rbfox2chb6 (this study), Tg(myl7:GFP-P2A-rbfox1l)chb7 (this study).

Wild animals

This study did not involve wild animals.

Field-collected samples

This study did not involve animals collected in the field.

Ethics oversight

Zebrafish were bred and maintained following protocols approved by the Institutional Animal Care and Use Committee (IACUC) of Massachusetts General Hospital and Boston Children's Hospital. All procedures followed the guidelines and recommendations outlined by the Guide for the Care and Use of Laboratory Animals.

Note that full information on the approval of the study protocol must also be provided in the manuscript.
