# Supplementary material for: Intrinsic myocardial defects underlie an Rbfox-deficient zebrafish model of hypoplastic left heart syndrome
Source: Nat Commun. 2022 Oct 5;13:5877. doi: 10.1038/s41467-022-32982-x (PMC9534849; doi:10.1038/s41467-022-32982-x)
Supplement: Supplementary file 3 — Description of Additional Supplementary Files [file 41467_2022_32982_MOESM3_ESM.docx]

**Description of Additional Supplementary Files**

File name: Supplementary Table 1

Description: Differential expression analysis of control and *rbfox1l; rbfox2* double mutant hearts.

File name: Supplementary Table 2

Description: Gene Ontology (GO) biological process terms derived from differential gene expression between control and *rbfox1l; rbfox2* double mutant hearts.

File name: Supplementary Table 3

Description: Alternative Splicing Events between control and *rbfox1l; rbfox2* double mutant hearts identified by rMATS.

File name: Supplementary Table 4

Description: Metabolomics data comparing profiles between 24 hpf control and *rbfox1l; rbfox2* double mutant embryos.

File name: Supplementary Table 5

Description: List of oligos/primers used in the study.

File name: Supplementary Movie 1

Description: Short movie of the heart beating in a control embryo carrying the Tg(myl7:GFP) transgene at 72 hpf.

File name: Supplementary Movie 2

Description: Short movie of the heart beating in an rbfox1l;rbfox2 double mutant embryo carrying the Tg(myl7:GFP) transgene at 72 hpf.
